# Targeting Tumor Vascular CD99 Inhibits Tumor Growth

**DOI:** 10.3389/fimmu.2019.00651

**Published:** 2019-04-02

**Authors:** Elisabeth J. M. Huijbers, Inge M. van der Werf, Lisette D. Faber, Lena D. Sialino, Pia van der Laan, Hanna A. Holland, Anca M. Cimpean, Victor L. J. L. Thijssen, Judy R. van Beijnum, Arjan W. Griffioen

**Affiliations:** ^1^Angiogenesis Laboratory, Department of Medical Oncology, Cancer Center Amsterdam, VU University Medical Center, Amsterdam UMC, Amsterdam, Netherlands; ^2^Hematology Laboratory, Department of Hematology, Cancer Center Amsterdam, VU University Medical Center, Amsterdam UMC, Amsterdam, Netherlands; ^3^Department of Histology, Angiogenesis Research Center Timisoara, Victor Babeş University of Medicine and Pharmacy, Timisoara, Romania

**Keywords:** CD99, MIC2, angiogenesis, tumor vasculature, vaccination, immunotherapy, cancer

## Abstract

CD99 (MIC2; single-chain type-1 glycoprotein) is a heavily O-glycosylated transmembrane protein (32 kDa) present on leukocytes and activated endothelium. Expression of CD99 on endothelium is important in lymphocyte diapedesis. CD99 is a diagnostic marker for Ewing's Sarcoma (EWS), as it is highly expressed by these tumors. It has been reported that CD99 can affect the migration, invasion and metastasis of tumor cells. Our results show that CD99 is also highly expressed in the tumor vasculature of most solid tumors. Furthermore, we found that *in vitro* CD99 expression in cultured endothelial cells is induced by starvation. Targeting of murine CD99 by a conjugate vaccine, which induced antibodies against CD99 in mice, resulted in inhibition of tumor growth in both a tumor model with high CD99 (Os-P0109 osteosarcoma) and low CD99 (CT26 colon carcinoma) expression. We demonstrated that vaccination against CD99 is safe, since no toxicity was observed in mice with high antibody titers against CD99 in their sera during a period of almost 11 months. Targeting of CD99 in humans is more complicated due to the fact that the human and mouse CD99 protein are not identical. We are the first to show that growth factor activated endothelial cells express a distinct human CD99 isoform. We conclude that our observations provide an opportunity for specific targeting of CD99 isoforms in human tumor vasculature.

## Introduction

The CD99 antigen, also known as MIC2 or single-chain type-1 glycoprotein, is a 32 kDa transmembrane protein expressed in inflamed endothelium and at low levels in thymocytes and T cells. Several reports show that CD99 is involved in lymphocyte diapedesis (1–6). Furthermore, CD99 has been presented as a diagnostic marker for Ewing's Sarcoma (EWS), as it is highly expressed by most EWS tumors (7, 8). In EWS tumors CD99 has been described to have an oncogenic function (9–13). Also, CD99 has been found to be involved in the migration, invasion and metastasis of tumor cells (5, 12, 14, 15).

Previous studies suggest that CD99 is a promising therapeutic target. Guerzoni et al. showed that targeting of CD99 by a diabody (C7 dAbd) promoted cancer cell death of EWS tumor cells *in vitro* (16). In addition, knockdown of CD99 in EWS tumor cells reduced *in vivo* tumor growth in mouse xenograft experiments (12). Also, a monoclonal antibody against CD99 (0662 Mab) combined with doxorubicin showed enhanced inhibition of EWS tumor growth and metastasis formation in a xenograft model (17). Imaging of mice with a ^64^Cu-labeled anti-mCD99 antibody detects subcutaneous Ewing sarcoma tumors and metastatic sites with high sensitivity (18). CD99 was also found to be highly expressed in acute lymphoblastic leukemia (ALL), acute myeloid leukemia (AML), myelodysplastic syndromes (MDS), and stem cells and anti-CD99 monoclonal antibodies show antileukemic activity in AML xenograft models (19–21). In atherosclerosis CD99 is expressed on activated endothelial cells that cover the plaque. Treatment of immunocompetent mice with an oral vaccine composed of attenuated *Salmonella typhimurium* transformed with pcDNA3-murineCD99 inhibited atherosclerotic plaque formation by induction of CD99 targeting cytotoxic T cells (22). Van Wanrooij et al. suggest that vaccination leads to removal of the CD99 expressing endothelial cells and thereby reduces atherosclerotic plaque formation. After vaccination a decreased expression of CD99 on leukocytes was observed and fewer leukocytes were recruited to the site of the plaque, whereas the total number of leukocytes was not affected. These observations indicate that CD99 can safely be used as a therapeutic target for vaccination.

In the current study, we explored the opportunity to use vaccination against CD99 as a treatment option against solid tumors. We showed that CD99 is heavily overexpressed on tumor endothelial cells in multiple human solid tumors. We developed a conjugate vaccine, based on a fusion protein technique published previously by Huijbers et al. (Supplementary Figure 1B) (23–25). In short, a fusion protein, consisting of the murine CD99 sequence and an engineered truncated version of bacterial thioredoxin (26), was made and used for vaccination. The vaccine induced an antibody response against CD99 by activation of specific CD99 auto-reactive B-cells. In two different immunocompetent mouse tumor models we found that vaccination against CD99 reduced tumor microvessel density and functionality, and resulted in suppressed tumor growth. No side-effects were observed after maintaining mice hyperimmune for almost a year.

For human CD99, two different isoforms have been described (27). A long full-length isoform (185 amino acids, 32 kDa, variant I; Supplementary Figure 1C) and a short truncated isoform (161 amino acids, 28 kDa, variant II), lacking most of the cytoplasmic domain, exist. The murine CD99 only shows 46% homology with human CD99 and resembles the human short isoform (28). However, it is unclear whether the CD99 isoforms have the same function in mouse as in humans (29). In the NCBI database six different protein coding human CD99 splice variants are suggested (Gene ID: 4267) (30). In this paper we dissected the expression of these splice variants in endothelial cells and EWS tumor cells. Our results show a difference in CD99 splicing in activated endothelial cells and EWS tumor cells, which provides opportunities for specific therapeutic targeting to treat cancer.

## Materials and Methods

### Cell Culture

The murine osteosarcoma cell line Os-P0107, derived from a spontaneous osteosarcoma arising in a female *VEGF*^*P*^*-GFP/C3H* transgenic mouse, was a kind gift of Dr. Dan Duda (Massachusetts General Hospital, Harvard Medical School, Boston, MA, USA) (31). The Os-P0107 cells were maintained in Dulbecco's modified eagle medium (DMEM) (cat no. BE12-604F, Lonza Biowhittaker, Leusden, The Netherlands) containing 15% Fetal bovine serum (FBS) (cat no. S1810-500, Biowest, Nuaillé, France) and 100 U/ml penicillin/streptomycin (pen/strep) (cat. no. DE17-602E Lonza Biowhittaker). CT26 murine colon carcinoma cells (CT26.WT) (ATCC no. CRL-2638, Manassas, VA, USA) were cultured in Roswell Park Memorial Institute (RPMI)-1640 supplemented with 2 mM L-glutamine (cat no. 17-605C, Lonza Biowhittaker) 10% FBS and 100 U/ml pen/strep.

The Ewing's Sarcoma cell lines EW7 and EWS-RDES were previously characterized by Dr. O. Delattre (Pathologie Moléculaire des Cancers, Institut Curie, Paris Cedex). Cells were maintained in RPMI-1640 supplemented with 10% FBS and 2 mM L-glutamine.

Human umbilical vein endothelial cells (HUVEC), isolated from umbilical cords by standard methods, were maintained in RPMI-1640 (Lonza Biowhittaker) medium supplemented with 10% FBS (Biowest), 10% human serum, 2 mM L-glutamine (Lonza Biowhittaker), and 100 U/ml pen/strep (Lonza Biowhittaker). HUVEC were cultured in 0.2% gelatin coated culture flasks.

### HUVEC Starvation Assay

Six-well culture plates (VWR International, Radnor, PA, USA) were coated with 0.2% gelatin and 150,000 HUVEC were seeded in each well. Cells were allowed to adhere for 3–4 h before nutrient deprived medium was added containing only 10% FBS or 1% FBS (Lonza Biowhittaker) in RMPI-1640 medium (Lonza Biowhittaker). Cells were then harvested at 24 or 48 h and flow cytometry was performed. In addition, cell lysates were prepared from control treated cells and 48 h starved cells. To this end cells grown in a T75 culture flask were scraped off the bottom in 500 μl 2x Laemmli sample buffer (#1610737, Bio-Rad Laboratories B.V., Veenendaal, The Netherlands) plus β-mercaptoethanol (sc-202966, Santa Cruz Biotechnology Inc., Dallas, TX, USA) on ice. Lysates were stored at −20°C until use.

### Flow Cytometry

HUVEC were harvested using trypsin/EDTA (Lonza Biowitthaker), washed with 0.1% BSA/PBS stained with rabbit anti-human CD99 polyclonal antibody (ab27271, 0.2 mg/ml, Abcam, Cambridge, UK) diluted 1:100 in 0.1% BSA/PBS for 30 min at RT. After a washing step cells were incubated with secondary anti-rabbit APC antibody (F0111, R&D Systems, Abingdon, UK).

Os-P0107 osteosarcoma and CT26 cells were collected with 0.5 M EDTA (Sigma Aldrich), centrifuged and resuspended in ice-cold PBS/10%FCS/0.1% sodium azide solution to prevent internalization of CD99. Cells were distributed into FACS tubes (1^*^10^6^ cells per tube), washed and centrifuged for 5 min at 400 g and 4°C in a Rotina 420R centrifuge (Hettich Lab Technology, Tuttlingen, Germany). To detect CD99 expression, cells were incubated in a volume of 50 μl with primary goat anti-mouse CD99 polyclonal antibody (1:100; R&D systems; AF3905, 0.2 mg/ml) in 0.1% BSA/PBS for 30 min at 4°C. Cells were washed and centrifuged three times before and after being incubated with donkey anti-goat IgG Northern Lights 557 (1:1,000; R&D systems; NL001) for 30 min at 4°C in the dark. Subsequently cells were resuspended in ice-cold PBS/10%FCS/0.1% sodium azide solution.

Cells were analyzed with a FACSCalibur flow cytometer (Beckton Dickinson, Franklin Lakes, NJ, USA) and CellQuest Software. Data analysis was performed with FCSalyzer (SourceForge, La Jolla, CA, US). Fold increase of the mean fluorescence intensity (MFI) was determined by dividing the MFI of CD99 stained cells by the MFI of control stained cells.

### Immunohistochemistry

Tumors and normal tissues were paraffin embedded and sectioned (5 μm) with a Leica RM 2135 microtome (Leica, Nieuw-Vennep, The Netherlands). Sections were dried overnight at 37°C, placed at 60°C for 1 h and baked for 10 min at 56°C before deparaffinization with xylene (VWR International) followed by 100% (Nedalco B.V., Bergen op Zoom, The Netherlands), 96 and 70% ethanol and rehydration in phosphate buffered saline (PBS). After treatment with 1% hydrogen peroxide (Hydrogen peroxide 30%, BDH Prolabo, VWR International, Amsterdam, The Netherlands) in PBS for 15 min at room temperature (RT), antigens were retrieved in 10 mM sodium citrate buffer pH 6.0. After cooling down, sections were washed in PBS and blocked with 3% Bovine Serum Albumin Fraction V (BSA, Roche Diagnostics, Penzberg, Germany)/PBS for 1 h at RT and incubated with rabbit anti-human CD99 polyclonal antibody (ab27271, Abcam) diluted 1:200 in 0.5% BSA/PBS overnight at 4°C. The next day, tissue sections were incubated with biotinylated swine anti-rabbit Ig antibody (E0353, 0.50 g/L, DakoCytomation, Glostrup, Denmark) diluted 1:500 for 45 min at RT. This was followed by incubation with Streptavidin-HRP (P0397, 0.82 g/L, DakoCytomation) diluted 1:200 in 0.5% BSA/PBS for 30 min at RT and 3,3′-diaminobenzidine tetrahydrochloride hydrate (DAB) staining (Sigma-Aldrich Chemie B.V., Zwijndrecht, The Netherlands).

Images of different tumor types and normal tissues stained for human CD99 were retrieved from the Human Protein Atlas (32, 33).

For staining of murine CD99, polyclonal goat anti-mouse CD99 antibody (AF3905, 0.2 mg/ml, R&D Systems,) diluted 1:50 was used. Endogenous peroxidase activity was blocked with 1% H_2_O_2_. After antigen retrieval with sodium citrate buffer, primary antibody was detected with biotinylated polyclonal rabbit anti-goat (E0466, 1.6 g/L, DakoCytomation) diluted 1:200 in 0.1% PBS-T, followed by Streptavidin-HRP (DakoCytomation) 1:400 in 0.1% PBS-T and DAB substrate. All sections stained with CD99 antibody, were counterstained with Mayer's hematoxylin (Klinipath, Duiven, The Netherlands) for 30 s and the reaction was stopped under running tap water for 10 min. Finally, sections were dehydrated in 70% ethanol, 96% ethanol and 100% ethanol for 2 min consecutively and mounted with Quick D mounting medium (Klinipath).

To determine vessel density and morphology, tumor tissue and organs, sections (3 μm) were stained for CD31 (34). To this end, cleared paraffin sections were incubated with 0.3% H_2_O_2_/PBS for 15 min at RT. Antigens were retrieved with sodium citrate buffer, slides were blocked with 3%BSA/PBS as described above and stained with rat anti-mouse CD31 antibody (DIA310M, clone SZ31, 0.2 mg/ml, Dianova GmbH, Hamburg, Germany), diluted 1:50 in 0.5% BSA/PBS overnight at 4°C. Tissue sections were subsequently incubated with biotinylated donkey anti-rat IgG antibody (product code: 712-067-003, 1.3 mg/ml, Jackson ImmunoResearch laboratories, Baltimore, PA, USA) diluted 1:500 in 0.5% BSA/PBS for 45 min at RT. Sections were washed in PBS and subsequently incubated with Streptavidin-HRP (1:200; DakoCytomation) in 0.5% BSA/PBS for 30 min at RT and developed with DAB substrate. Finally, sections were dehydrated in 70% ethanol, 96% ethanol, and 100% ethanol for 2 min consecutively and mounted with Quick D mounting medium (Klinipath).

### Desmin Staining

After deparaffinization, osteosarcoma tumor sections were treated with 0.3% H_2_O_2_ for 15 min at RT, washed and boiled in citrate buffer. Sections were blocked with 3% BSA/PBS for 60 min at RT and incubated with primary rat anti-mouse CD31 antibody (1:50; Dianova) and goat anti-human/mouse desmin antibody (1:200; R&D systems; AF3844) in 3.0% BSA/PBS overnight at 4°C. Primary antibody was detected with secondary rabbit anti-rat HRP (1:100; DakoCytomation; P045001) and rabbit anti-goat biotinylated (1:200; DakoCytomation) in 3.0% BSA/PBS for 30 min at RT. After a washing step, sections were developed with DAB substrate to detect the CD31 staining. To detect the pericytes, sections were washed and incubated with strep-AP (1:500; Genmed Synthesis Inc. San Antonio, Texas, USA) in 10X TBS Solution (0.5 M Tris-Cl; 1.5 M NaCl, pH 7.6) for 30 min at RT. Washed in ddH_2_O and developed with Fast Blue BB (Sigma Aldrich). Finally, sections were washed in ddH_2_O, air-dried and embedded in Kaisers glycerol gelatin (Merck group).

### Staining of Tumor Tissue With Serum of TRXtr-mCD99 Immunized Mice

Osteosarcoma tumor sections from TRXtr immunized mice were deparaffinized, treated with 1% H_2_O_2_ for 15 min at RT and boiled in citrate buffer. Sections were blocked with 20% horse serum (H-1138, heat inactivated, Sigma-Aldrich)/PBS for 1 h at RT. Consecutively, sections were incubated with goat F(ab) anti-mouse IgG H&L (ab6668, 1 mg/ml, Abcam) diluted 1:20 in PBS for 2 h at RT, to prevent non-specific binding of the mouse serum to the mouse tissue. A washing step with 0.05% PBS-T was performed and the sections were stained with serum from TRXtr immunized or TRXtr-mCD99 immunized mice diluted 1:600 in 20% horse serum/PBS overnight at 4°C. Anti-CD99 antibodies in the serum were detected with biotinylated polyclonal goat anti-mouse Ig (E0433, Dako Cytomation) diluted 1:500 in 0.5% BSA/PBS, followed by Streptavidin-HRP (DakoCytomation) 1:200 in 0.5% BSA/PBS and DAB substrate. Sections were counterstained with Mayer's hematoxylin (Klinipath), dehydrated in an ethanol series and mounted with Quick D (Klinipath).

### Hematoxylin/Eosin Staining

Sections were deparaffinized and dipped in diluted Mayer's Hematoxylin (Klinipath) (1:4 dilution in 5 mM sodium citrate buffer pH 6.0). After a rinse under flowing tap water for 5 min, sections were stained with 0.2% eosin Y solution (J.T. Baker, Avantor Performance Materials B.V., Deventer, The Netherlands) for 30 s. Sections were dehydrated with two changes of 70% ethanol, three changes of 96% ethanol, 100% ethanol for 5 min and xylene for 2 min. Consecutively sections were mounted with Quick D mounting medium (Klinipath).

### Immunohistochemistry Quantification

Pictures were captured with an Olympus BX50 microscope (Olympus Optical Co. GmbH, Hamburg, Germany) equipped with a CMEX DC 5000C camera (Euromex microscopes, Arnhem, The Netherlands).

Only viable tumor tissue was used for analysis. Microvessel density was assessed by manual counting of tumor tissue stained for CD31. In total 3 fields/tumor (100x magnification) and 3–10 tumors per experimental group were counted. Images were used to manually count the number of vessels with a clear lumen in osteosarcoma tumors. Images were further analyzed with ImageJ (Laboratory for Optical and Computational Instrumentation, University of Wisconsin-Madison; Version 1.51s) to determine the vessel density of osteosarcoma and CT26 tumors. For pericyte (desmin) quantification, 10 fields per tumor were chosen (magnification 200x). Images were used to manually count the number of vessels with and without desmin staining/associated pericytes. Pericyte coverage was then determined by dividing the number of vessels with pericytes by the total vessel count.

### Reverse Transcriptase Quantitative Polymerase Chain Reaction (RT-qPCR)

Total RNA was isolated using TRIzol Reagent (Life technologies, Carlsbad, CA, USA) according to the manufacturer's protocol. RNA concentration and quality were measured using a NanoDrop ND-1000 spectrophotometer (Isogen Life Science, Utrecht, The Netherlands). One microgram of the obtained RNA was reverse transcribed to cDNA using an iScript cDNA synthesis kit (Bio-Rad Laboratories, Hercules, CA, USA). The obtained cDNA was diluted 1:5 and RT-qPCR was performed using iQ SYBR Green Supermix (Bio-Rad Laboratories) and 0.2 μM of each primer (Eurogentec, Seraing, Belgium) (Tables 1, 2). Primers were validated as previously described (35). Samples were run in duplicate and analyzed on the CFX96 Real Time System C1000 Thermal Cycler (Bio-Rad Laboratories). Data were analyzed with CFX Manager software (version 3.1, Bio-Rad Laboratories), and further processed in MS Excel. All samples were normalized to cyclophillin A, β-actin, and β2-microglobulin transcript expression (Tables 3, 4) to account for variations in template input (35). The following formula was used to calculate the 2^−dCt^ value of the gene of interest: 2^−(Ctvaluegeneofinterest−meanCtvaluereferencegenes)^. Ratios of the different primer pairs and the k + l primers used to determine total CD99 were calculated by dividing the mean 2^−dCt^ value of each primer pair with the mean 2^−dCt^ value of the k + l primers.

**Table 1:** Primers RT-qPCR endogenous mCD99 in mouse cell lines.

**Primer**	**Sequence**
mCD99fw	5′-GCCTCGCCTGAATATGCAAA-3′
mCD99rev	5′-GTCAGTTGTGGGCGGAGTCTT-3′

**Table 2:** RT-qPCR Primers hCD99 isoforms.

**Primer**	**Sequence**
a	5′-TGCTGCTCTTCGGCCTGCTG-3′
b	5′-TTCTTGGGGATTGCAGTGGG-3′
c	5′-TCAAAGTCATCCCCAGGAAGG-3′
e	5′-TTGGCATTCCGGTGGCTCTC-3′
f	5′-GTTTCAGGTGGAGAAGCCGAC-3′
g	5′-TGATCCCCGGGATTGTGG-3′
h	5′-TTCTCTGCTCACCCCTAGGTC-3′
k	5′-AATGACGACCCACGACCACC-3′
l	5′-AACGCCATCCGCAAGGTCAG-3′

**Table 3:** Mouse RT-qPCR reference gene primers.

**Primer**	**Sequence**
β-actin fw	5′- GAAGCTGTGCTATGTTGCTCTA−3′
β-actin rev	5′- GGAGGAAGAGGATGCGGCA−3′
β-2microglobulin fw	5′- CCGCCTCACATTGAAATCC−3′
β-2microglobulin rev	5′- CTCTGCAGGCGTATGTATCAG−3′
Cyclophilin A	5′- ATTTCTTTTGACTTGCGGGC−3′
Cyclophilin A	5′- AGCTAGACTTGAGGGGAATG−3′

**Table 4:** Human RT-qPCR reference gene primers.

**Primer**	**Sequence**
β-actin fw	5′- CATTCCAAATATGAGATGCATT−3′
β-actin rev	5′- CCTGTGTGGACTTGGGAGAG−3′
β-2microglobulin fw	5′- TCGATCCGACATTGAAGTTG−3′
β-2microglobulin rev	5′- ACACGGCAGGCATACTCAT−3′
Cyclophilin A	5′- CTCGAATAAGTTTGACTTGTGTTT−3′
Cyclophilin A	5′- CTAGGCATGGGAGGGAACA−3′

### Expression Vectors

The region encoding the extracellular part [amino acids (aa) 27–137] of murine CD99 (36) (GenBank TM/EBI Data Bank accession numbers BC019482) (UniProtKB-Q8VCN6 (CD99_mouse) (RefSeq NP_079860.2. NM_025584.2.; GI: 125660452), optimized for protein expression in bacteria (Genscript) (333 bp), was inserted downstream the bacterial truncated thioredoxin (TRXtr) (26) sequence (192 bp) containing an N-terminal 4xGS-linker sequence and His-tag (6xhistidine) into a pET21a (+) vector (Novagen; EMD Chemicals, Gibbstown, NJ, USA). The original pET21-TRX plasmid was a kind gift of Dr. Anna-Karin Olsson (Uppsala University, Uppsala, Sweden). The resulting expression vector was named pET21a-TRXtr-mCD99_extracellular_ (Supplementary Figure 1D).

Murine CD99 extracellular part protein sequence (aa27-137) (36): ASDDFNLGDALEDPNMKPTPKAPTPKKPSGGFDLEDALPGGGGGGAGEKPGNRPQPDPKPPRPHGDSGGISDSDLADAAGQGGGGAGRRGSGDEGGHGGAGGAEPEGTPQ

For construction of the pET21a-mCD99_extracellular_ plasmid the extracellular part of murine CD99 was PCR amplified from the original pET21a-mCD99 vector (Genscript) using the following primers:

Nde1-His-extracellmCD99fw
  5′-TAT-CAT-ATG-CAC-CAC-CAC-CAC-CAC-CAC-GCA-AGC-GAT-GAT-TTT-A-3′
Xho1-3x-stop-excellmCD99rev
  5′-TAT-CTC-GAG-CTA-TTA-TCA-ACC-CTG-CGG-GGT-ACC-TTC-CGG-TTC-3′

After purification and restriction with *NdeI* and *XhoI* the mCD99_extracellular_ sequence was ligated into a pET21a vector.

### Vaccine Production and Purification

**Figure 1:**
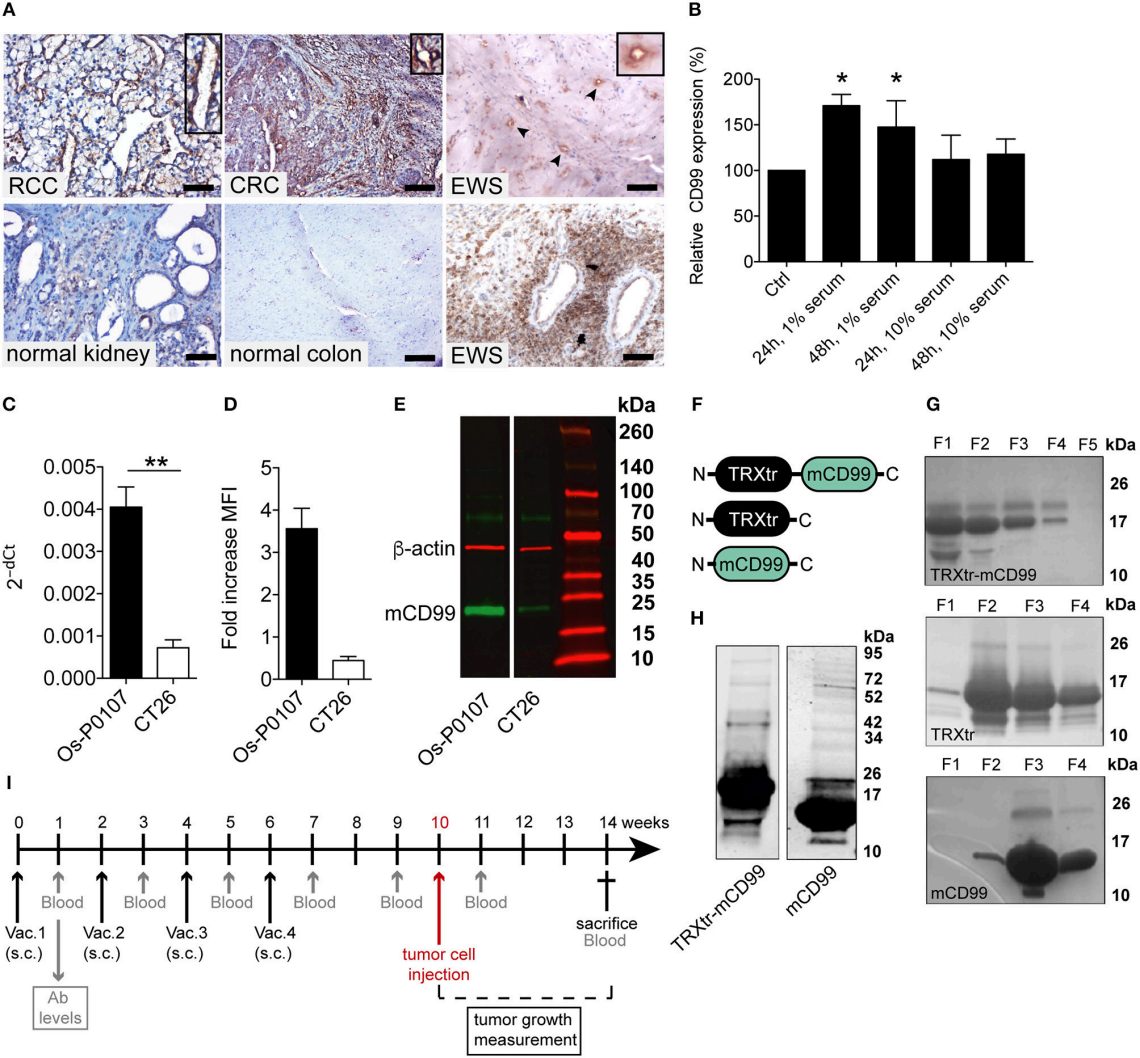
CD99 expression in the vasculature of solid tumors and in the mouse tumor cell lines used; purification of vaccine proteins. **(A)** Tumor and normal tissue stained for CD99. Clear expression of CD99 in the tumor vasculature can be observed in renal cell carcinoma (RCC), colorectal cancer (CRC) and Ewing sarcoma (EWS) (upper row). Inlays show enlargement of CD99 staining in the tumor vessels. Normal healthy kidney and colon are devoid of CD99 (lower left panel and lower middle panel). In EWS both tumor cells (lower right panel) and the tumor endothelium (upper right panel, arrow heads and inlay) express CD99. (EWS, RCC, normal kidney: scale bar 50 μm; CRC and normal colon: scale bar 35 μm). **(B)** Expression of CD99 is increased in serum starved HUVEC. HUVEC were cultured for 24 or 48 h under normal conditions in culture medium supplemented with 20% serum (control, Ctrl) or in medium supplemented with only 1 or 10% serum (*n* = 3). CD99 expression was determined by flow cytometry analysis of non-fixated cells. Means were compared using a Mann-Whitney U test (^*^P < 0.05). **(C)** Relative expression (2^−dCt^) of mouse CD99 mRNA in Os-P0107 (black bar) and CT26 tumor cells (white bar); ^**^P < 0.01. **(D)** FACS analysis of CD99 surface expression in Os-P0107 and CT26 tumor cells. **(E)** Western blot analysis of total cell lysates of Os-P0107, and CT26 cells stained with antibodies against murine CD99 (24–30 kDa) and actin (42 kDa). Both Os-P0107 and CT26 cells show clear CD99 protein expression. CT26 cells have low levels of CD99. **(F)** Schematic representation of the recombinant fusion protein TRXtr-mCD99 used for vaccination of mice and the proteins TRXtr (used for vaccination of control mice) and mCD99 used to measure anti-CD99 antibodies in the sera of the vaccinated mice. **(G)** Appearance of TRXtr-mCD99 (18 kDa), TRXtr dimer (15 kDa) and mCD99 (11 kDa) proteins on reducing SDS-PAGE after elution from the Ni-NTA agarose with imidazole. For each protein all eluted fractions **(F)** are shown. **(H)** Western blot of TRXtr-mCD99 and mCD99 to confirm protein identity. **(I)** Illustration of the experimental set-up. Mice are vaccinated four times (Vac.; black arrows). At set time-points blood samples are taken for measurement of anti-mCD99 antibodies in the sera of the mice (blood; gray arrows). When anti-mCD99 levels are high, tumor cells are injected subcutaneously into the left flank of the mice (red arrow). Tumors are allowed to grow 3–4 weeks before the mice are sacrificed (cross).

The recombinant vaccine proteins were produced and purified as previously described (23, 24). The pET21a expression vectors were transformed into *E. Coli* Rosetta gami (DE3) (Novagen; Merck Millipore, Darmstadt, Germany) for recombinant protein expression. Overnight cultures were diluted 1:3 and were grown until optical density 600 nm (OD_600_) 0.5 was reached. Protein expression was induced with 1 mM isopropyl β-D-1-thiogalactopryanoside (IPTG, Invitrogen, Life Technologies, CA, USA) at 37°C for 4 h for TRXtr-mCD99_extracellular_ and mCD99_extracellular_ (for simplicity the resulting proteins are named TRXtr-mCD99 and mCD99). TRXtr expression was induced at 22°C for 15 h. Bacteria were harvested by centrifugation at 4,500 rpm (3,584 g), 10 min, 4°C (Hettich Rotina 420R) and washed five times with PBS. Bacterial pellets were dissolved in PBS (TRXtr-mCD99 and mCD99) or in 5 M urea (TRXtr) (Acros Organics/Thermo Fisher Scientific, Landsmeer, The Netherlands). The proteins (TRXtr-mCD99 and mCD99) were released by sonication (amplitude 22–26 microns, Soniprep 150 MSE, London, UK) on ice, 12 times for 30 s with breaks of 30 s, for the TRXtr protein 15 cycles of 20 s on and 30 s off were used. After centrifugation, 20 mM imidazole (J.T. Baker, Avantor Performance Materials B.V.) was added to the supernatant to reduce non-specific binding of background proteins to the nickel (Ni) agarose. No imidazole was added to the TRXtr supernatant during the Ni-agarose incubation step. Thereafter, 500 μl 50% Ni-NTA agarose slurry (Qiagen, Venlo, The Netherlands) was mixed with 25 ml supernatant (originating from 500 ml bacteria culture) and incubated “end-over-end” at 4°C for 3 h. After centrifugation at 3,000 rpm (Rotina 420R, Hettich), the agarose beads were washed with 250 ml wash buffer containing PBS pH 7.0/1 M NaCl /0.1% Tween-20. An additional washing step with PBS was performed to remove the detergent Tween (P1379, Sigma-Aldrich, Zwijndrecht, The Netherlands). Then, the beads were transferred to a 1 ml syringe (BD Plastipak, BD Biosciences, Madrid, Spain) with a glass filter (Sartorius Stedim Biotech, Göttingen, Germany) and washed again with PBS. The TRXtr-mCD99 protein was eluted with two 250 μl fractions of 50 mM and three fractions of 100 mM imidazole, dissolved in 20 mM Tris pH 8.0/0.1 M NaCl. The TRXtr and mCD99 protein were eluted with four 250 μl fractions of 100 mM imidazole solution. Protein content of the separate fractions was determined by SDS-PAGE using precast 4–20% gradient polyacrylamide gels (Mini-Protean TGX, Bio-Rad Laboratories). Gels were fixed and stained with home-made colloidal Coomassie brilliant blue R250 solution containing 20% methanol (VWR International). Destaining of the gels was performed with methanol for 1 min and ddH_2_O for several hours. Fractions containing most protein were pooled and dialyzed against PBS (pH 7.0). The different recombinant proteins TRXtr-mCD99 (18 kDa), TRXtr (7.5 kDa; appears as a 15 kDa dimer on reduced SDS-PAGE) and mCD99 (11 kDa) as present on an SDS-PAGE gel after purification (Figure 1G).

For the TRXtr and TRXtr-mCD99 protein a Slide-A-Lyzer Dialysis cassette (M_w_ cut-off (MWCO) 7,000 Da; Thermo Fisher Scientific) was used. The mCD99 protein was dialyzed in snakeskin dialysis membrane (MWCO 3,500 Da; Thermo Fisher Scientific). Final protein concentrations were measured by micro BCA protein assay (Pierce Biotechnology, Rockford, IL, USA).

### Production of Rosetta Gami Extract to Block Background Binding in ELISA

Rosetta gami extract for use in ELISA was produced from uninduced pET21a-TRX transformed overnight cultures. Bacteria were harvested at 4,500 rpm, 10 min, 4°C (Rotina 420R, Hettich) and washed 3 times with PBS. The pellet (originating from 200 ml overnight culture) was resuspended in 10 ml 0.5 M urea and sonicated for 15 cycles 20 s on and 30 s off (amplitude 22–26 microns, Soniprep 150 MSE). Bacterial lysates were centrifuged at 4,500 rpm, 10 min, 4°C (Rotina 420R, Hettich) and supernatants saved at −20°C until use.

### Western Blot

Cell lysate of Os-P0107 and CT26 was obtained using RIPA buffer (Cell Signaling Technology, Danvers, MA, USA) with the addition of HALT protease/phosphatase inhibitor 1:100 (Thermo Fisher Scientific) and stored at −20°C until use. Protein concentration was measured by Nanodrop ND-1000 spectrophotometer (Isogen Life Science).

Samples were separated by SDS-PAGE on a precast 4–20% gradient polyacrylamide gel (Mini-Protean TGX gel, Bio-Rad Laboratories). Thereafter, proteins were transferred/blotted to a methanol activated nitrocellulose membrane [Immobilon® PVDF (polyvinylidene) membrane (Merck Millipore, Darmstadt, Germany)] in a Bio-Rad blotting system with transfer buffer (500 mM Glycine, 50 mM Tris-HCl, 0.01% SDS, 20% methanol; Bio-Rad) at 100 V for 2 h on ice. The membrane was washed with 0.05% Tween-20/PBS and blocked with 5% BSA (Roche, Woerden, The Netherlands)/PBS-T 0.05% for 1 h before incubation with the primary antibody, goat polyclonal anti-mouse CD99 (AF3905, R&D Systems) 1:1,000 in 1% BSA/PBS-T 0.05% overnight at 4°C. The next day the membrane was incubated with the secondary antibody, biotinylated rabbit polyclonal anti-goat antibody (E0466, 1.6 g/l Dako Cytomation) 1:600 in 1% BSA/PBS-T 0.05% for 45 min followed by Streptavidin IRDye 800CW (cat no. 926-32230, LI-COR Biosciences, Lincoln, NE, USA) 1:10 000 in 1% BSA/PBS-T 0.05% for 30 min, both at room temperature in the dark. After this step, the blot was washed overnight in PBS-T 0.05% and the next day incubated with rabbit anti-mouse β-actin antibody (cat no. 49675, Cell Signaling Technology, Leiden, The Netherlands), dilution 1:1,000, for 1 h at RT and donkey anti-mouse IRDye 680RD antibody (cat no. 925-68072, LI-COR Biosciences) diluted 1:10 000 for 30 min at RT. After each incubation step membranes were washed with 0.05% Tween-20/PBS and with PBS prior to imaging with the Odyssey Infrared Imaging System (Model 9120, LI-COR Biosciences).

For staining of human CD99 the membrane was blocked with 5% BSA/PBS-T 0.05% and incubated with rabbit anti-human CD99 polyclonal antibody (ab27271, Abcam) diluted 1:200 in 1% BSA/PBS-T 0.05% overnight at 4°C. The next day the membrane was incubated with goat anti-rabbit IRDye 800CW antibody (cat no. 926-32211, LI-COR Biosciences) diluted 1:10,000 in 1% BSA/PBS-T 0.05%. Washing steps were performed as described above. The blot was stored overnight in PBS and blocked the next day with 5% non-fat dry milk (blotting-grade blocker, cat no. 170-6404, Bio-Rad Laboratories)/PBS-T 0.1%. After that the membrane was stained overnight at 4°C with mouse monoclonal anti-human β-actin antibody (cat no. #3700, clone 3H10D10, Cell Signaling Technology, Leiden, The Netherlands) diluted 1:1,000 in 1% non-fat dry milk/PBS-T 0.1%. As final step the membrane was incubated with donkey anti-mouse IRDye 680D (cat no. 925-6872, LI-COR Biosciences) diluted 1:10,000 in 1% non-fat dry milk/PBS-T 0.1%. All washing steps were performed with PBS-T 0.1% and performed as described above. The membrane was imaged with the Odyssey Infrared Imaging System (LI-COR Biosciences).

### Animal Studies

Animal experiments were approved by the local Animal Ethics Committee of the VU University and the national Central Animal Experiments Committee (CCD) (reg. no. AngL14-01 and CCD AVD114002016576). Approximately 8-week old female C3H/HeNCrL mice (Charles River Laboratories, Leiden, The Netherlands) or BALB/c mice (Envigo, Horst, The Netherlands) were immunized four times with an interval period of 2 weeks. Each vaccine emulsion (100 μl per mouse, 50 μl per groin) contained 40 μg TRXtr (control group) or 100 μg TRXtr-mCD99 in a volume of 50 μl mixed with 50 μl Freund's complete adjuvant (F-5881, Sigma Aldrich) (ratio 1:1, aqueous phase: oil phase) for the priming immunization and Freund's incomplete adjuvant (F-5506, Sigma Aldrich) for booster immunizations. Emulsions were mixed for 30 min on a Vortex Genie (Fisher Scientific) at full speed. Four weeks after the last immunization 2 × 10^6^ osteosarcoma (Os-P0107) cells were inoculated subcutaneously in the left flank of C3H mice in a total volume of 100 μl (10% culture medium/PBS). For the CT26 model 2 × 10^5^ CT26 colon carcinoma cells were inoculated in the left flank of BLALB/c mice. Blood samples were taken from the tail vein 1 week after each immunization, 1 week prior to tumor cell injection, and 1 week after tumor cell injection and at the end of the experiment. Tumor growth was measured by calipers. Tumor volume was calculated by the formula: width^2^ × length × π/6. At the end of the experiment mice were euthanized and tumors and organs were removed and stored in 1% PFA/PBS (Aurion, Wageningen, the Netherlands) overnight and consecutively paraffin embedded.

### Long-Term Follow-Up After CD99 Vaccination

To address the safety of exposure to high antibody titers against CD99; control vaccinated (TRXtr, *n* = 5) and TRXtr-mCD99 vaccinated mice (CD99; *n* = 5) were included in the study for 45 weeks. Approximately 8-week old female C57BL/6 mice (Envigo) were immunized three times with an interval period of 2 weeks as described above. Blood samples were taken from the tail vein 1 week after each immunization. During the rest of the follow-up period monthly blood samples were taken. When antibody levels dropped below 50% of the levels after the third vaccination mice were revaccinated. In addition, body weight of the mice was monitored regularly during the whole study period. At the end of the experiment mice were euthanized and organs were removed, stored in 1% PFA/PBS (Aurion) overnight and paraffin embedded.

### ELISA

Indirect ELISA was performed to determine total anti-mCD99 antibody levels. Blood samples were coagulated overnight at 4°C and centrifuged twice at 7,000 rpm for 10 min at 4°C in a microcentrifuge. The supernatant (serum) was stored at −20°C until use. Volumes used per well in ELISA were 50 μl, unless indicated otherwise. 96-well ELISA plates (Nunc A/S, Roskilde, Denmark) were coated with 2 μg/ml mCD99 protein and then blocked with 100% horse serum (100 μl/well) (Sigma-Aldrich), both for 1 h at 37°C. After a single wash with PBS (B. Braun Medical, Oss, The Netherlands) for 1 min, the plates were incubated with serum of TRXtr-mCD99 or TRXtr-vaccinated mice for 45 min at 37°C, diluted 1:10 in 100% horse serum, which was further diluted 1:15 in 10% Rosetta Gami extract (final serum dilution 1:150) to reduce non-specific binding of the serum. Thereafter, plates were incubated with biotinylated polyclonal goat anti-mouse Ig (E0433, Dako Cytomation) for 45 min and streptavidin-horseradish peroxidase (Strep-HRP) (Dako Cytomation) for 30 min, both diluted 1:2,000 in 0.01% PBS-T at 37°C. After each incubation step, plates were washed four times with PBS. HRP activity was detected with TMB substrate (T-8665, Sigma-Aldrich) and absorbance was measured at 655 nm after 15 min using a Biotek Synergy HT microplate reader (Biotek).

### Statistical Analysis

Means were compared using a Mann–Whitney *U*-test or two-tailed student's *t*-test, if Gaussian distribution could be assumed. For comparison of tumor growth, a two-way ANOVA with Bonferroni post-test was used for repeated measurements at different time points. Values are depicted as mean ± SEM. All statistical tests were executed using GraphPad Prism 5.0 (GraphPad Software Inc., La Jolla, CA, USA). Values of *P* < 0.05 were considered statistically significant.

## Results

### CD99 Is a Marker of Tumor Blood Vessels and Is Induced by Starvation

The Ewing's sarcoma (EWS) tumor marker CD99 was found to be overexpressed in the vasculature of different solid tumor types (Figure 1A; Supplementary Figure 1A), but CD99 was not expressed in normal healthy tissues. In endothelial cells, cultured *in vitro*, CD99 expression was upregulated upon 24 and 48 h of starvation (*p* < 0.05) (Figure 1B).

### Expression of CD99 in Murine Tumor Cell Lines

In order to determine the CD99 expression in the murine osteosarcoma (Os-P0107) and colon carcinoma (CT26) cell lines qPCR primers were designed to determine the expression of endogenous CD99 (mCD99; RefSeq NP_079860.2. NM_025584.2.; GI: 125660452) (Table 1). Expression of CD99 at the mRNA level was confirmed in both cell lines (Figure 1C). The CT26 cell line was observed to express significantly lower levels of CD99 than the Os-P0107 cell line [(^**^P < 0.01); Figure 1C, white bar]. At the protein level, flow cytometry was used to check for surface expression of CD99. These data indicated a higher surface expression of CD99 on Os-P0107 cells compared to the CT26 cell line (Figure 1D). In order to clearly distinguish any difference in CD99 protein level between the two cell lines we performed a Western blot analysis (Figure 1E) on cell lysate. Indeed, the Western blot confirmed that the osteosarcoma cells express high levels of murine CD99 (24 kDa, green band) and that CD99 expression in the CT26 cell line is much lower. β-actin (42 kDa, red band) was used as a loading control. In addition, the ratio of the CD99 and β-actin bands was quantified with ImageJ software and the osteosarcoma cell line showed a higher CD99/β-actin ratio than the CT26 cell line (Os-P0107 ratio: 1.77; CT26 ratio: 0.46) (Table 5).

**Table 5:** Western blot quantification CD99 expression cell lines.

	**Band area CD99**	**Band area b-actin**	**Ratio CD99/b-actin**
Os-P0107	6045.447	3411.870	1.77
CT26	876.648	1891.920	0.46

### Design/Construction of a CD99 Targeting Vaccine

The overexpression of CD99 in human tumor vasculature prompted us to study the role of the molecule in tumor growth. To that end, we planned to vaccinate against CD99, using the conjugate vaccine technology that we recently published (26). The extracellular part of murine CD99 (mCD99_extracellular_, 111aa; RefSeq NP_079860.2. NM_025584.2.; GI: 125660452) (36) was used as the self-antigen to be fused to truncated bacterial thioredoxin (TRXtr, 58aa) (26), resulting in the fusion protein TRXtr-mCD99 (Figure 1F).

The TRXtr protein was produced for vaccination of control mice and mCD99 for detection of anti-mCD99 antibodies in serum by ELISA (Figure 1F). All proteins were soluble (Figure 1G). Protein identity of TRXtr-mCD99 and mCD99 was confirmed by Western blot analysis (Figure 1H).

### Vaccination Against CD99 Inhibits Tumor Growth

**Figure 2:**
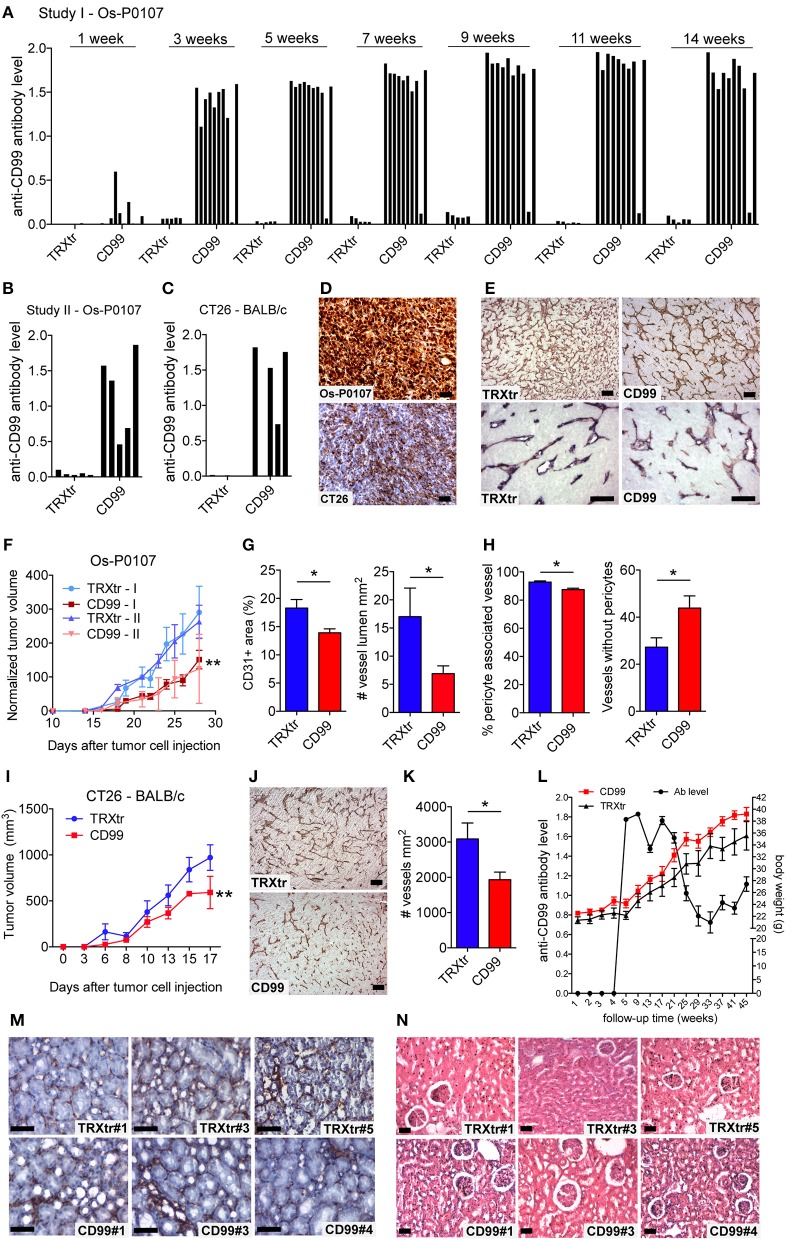
Induction of a humoral immune response against CD99 inhibits tumor growth and is safe. **(A)** Anti-mCD99 antibody levels in the sera of the C3H mice (Os-P0107 model) at different time points (weeks) (1st study, Study I; *n* = 10, CD99). In the sera of TRXtr vaccinated mice (*n* = 5, TRXtr, control) no anti-mCD99 antibodies were present. **(B)** Anti-mCD99 antibody levels in the sera of CH3 mice at time point week 9 (after four vaccinations, prior to tumor cell injection) (2nd study, Study II; *n* = 5, CD99). The control vaccinated mice (*n* = 5, TRXtr) did not have any anti-mCD99 antibodies. **(C)** Anti-mCD99 antibody levels in the sera of the BALB/c mice (CT26 model) after four vaccinations (week 7), prior to tumor cell injection. TRXtr-mCD99 vaccinated mice (*n* = 4, TRXtr-mCD99) responded with the production of anti-mCD99 antibodies, whereas in the sera of TRXtr vaccinated mice (*n* = 5, TRXtr, control) no anti-mCD99 antibodies were present. **(D)** Immunohistochemistry staining of CD99 of Os-P0107 (upper panel) and CT26 tumors (lower panel) (8 μm scale bar). CD99 expression in Os-P0107 is higher than in CT26 tumors. **(E)** Staining of the tumor vasculature (CD31) of Os-P0107 tumors of control vaccinated (TRXtr, left upper panel, scale bar 35 μm) and TRXtr-CD99 vaccinated mice (CD99, right upper panel, scale bar 35 μm). Lower panels show double staining of the tumor vasculature with CD31 (red) and desmin (blue), a pericyte marker (scale bars 50 μm). **(F)** Tumor growth curves of Os-P0107 in TRXtr-mCD99 vaccinated (CD99; red curves) and control vaccinated mice (TRXtr; blue curves). In both studies (Study I and II) tumor growth was significantly inhibited in TRXtr-mCD99 vaccinated compared to control vaccinated mice. Tumor volume was normalized and growth curves were compared by two-way ANOVA (^**^P < 0.01). **(G)** Vessel density of osteosarcoma tumor tissue [CD31+ area (%)]. Vessel density was determined of 3 representative fields per tumor (magnification 100x). Tumors from TRXtr-mCD99 vaccinated mice (CD99; *n* = 9; red bars) had a lower vessel density compared to control vaccinated mice (TRXtr; *n* = 4; blue bars), (left panel, ^*^P < 0.05). The number of clear lumina found per field of osteosarcoma tumor (Os-P0107) tissue (magnification 100x, 3 fields per tumor). Mice vaccinated with TRXtr-mCD99 (CD99) had a significantly lower lumen count compared to control vaccinated mice (TRXtr) (right panel, ^*^P = 0.05). **(H)** Percentage of vessels associated with pericytes in osteosarcoma tumors. The number of vessels with and without pericytes was manually counted of 10 fields per tumor (magnification 200x). The number of vessels associated with pericytes was divided by the total number of vessels per tumor. Tumors of TRXtr-mCD99 vaccinated (CD99, red bars) mice had a significantly lower pericyte coverage compared to control vaccinated (TRXtr, blue bars) tumor tissue (left panel, ^*^P < 0.05). In addition, in tumors of TRXtr-mCD99 vaccinated mice significantly more vessels were found without pericytes than in tumors of control vaccinated mice (right panel, ^*^P < 0.05). **(I)** Tumor growth curves of CT26 in TRXtr-mCD99 vaccinated (CD99; red curve) and control vaccinated BALB/c mice (TRXtr; blue curve). Vaccination against CD99 significantly inhibited tumor growth in the CT26 tumor model as well (two-way ANOVA; ^**^P < 0.01). **(J)** Representative images of CD31 stained CT26 tumors of control vaccinated mice (TRXtr, upper panel) and TRXtr-mCD99 vaccinated mice (CD99, lower panel) (scale bars 35 μm). **(K)** Vessel density of CT26 tumor tissue. Vessel density was determined of 3 fields per tumor (magnification 100x). Significantly fewer vessels were counted in tumors of TRXtr-mCD99 vaccinated mice (CD99; *n* = 4; blue bar) compared to control vaccinated mice (TRXtr; *n* = 3; red bar) (^*^P < 0.05). **(L)** Vaccinated mice with high anti-CD99 antibody levels in their blood (antibody level is shown on the left y-axis; curve with marked black circles) were followed-up for a period of 45 weeks. No difference in body weight (right y-axis) between TRXtr-mCD99 vaccinated (CD99, red curve, squares) and control vaccinated mice (TRXtr, black curve, triangles) was found between the groups. Values are depicted as mean ± SEM. **(M)** Kidneys stained for CD31 (brown-reddish staining) of TRXtr-mCD99 (CD99) and control vaccinated (TRXtr) mice from the long-term follow-up study (time point 45 weeks). Tissues were counter stained with Mayer's hematoxylin (blue) (scale bar 50 μm). No difference in tissue morphology was found between TRXtr-mCD99 vaccinated and control vaccinated mice. **(N)** Hematoxylin eosin staining of kidneys of TRXtr-mCD99 (CD99) and control vaccinated (TRXtr) mice from the long-term follow-up study (time point 45 weeks) (scale bar 35 μm). No difference in tissue morphology was observed between the CD99 and TRXtr group, indicating that vaccination against CD99 is safe.

C3H mice were vaccinated with TRXtr-mCD99 (CD99; *n* = 10) or TRXtr (control group; *n* = 5). At week 10, when the mice were hyperimmune and had high antibody titers against murine CD99 in their sera, osteosarcoma (Os-P0107) tumor cells were injected into the left flank (see experimental set-up Figure 1I). Blood samples were collected 1 week after each vaccination, 1 week prior to tumor cell injection, 1 week after tumor cell injection and at the end of the experiment. In two independent experiments the mice responded with the production of anti-mCD99 antibodies (Figures 2A,B; Supplementary Figure 2A,B). Indeed, when antibodies against CD99 were present in the sera of the mice, tumor growth of osteosarcoma (Os-P0107) was significantly inhibited compared to control vaccinated mice (^**^P < 0.01; Figure 2F). We also investigated if tumor growth of the CD99 low CT26 colon carcinoma (Figure 2D, lower panel) could be inhibited after vaccination against CD99. All TRXtr-mCD99 (CD99 group; *n* = 5) vaccinated BALB/c mice responded with the production of anti-mCD99 antibodies (Figure 2C, one mouse died of unrelated cause). Also, in this study a significant difference in tumor growth between the TRXtr-mCD99 group and control group (TRXtr group; *n* = 5) could be observed (^**^P < 0.01; Figure 2I), indicating that in this tumor model inhibition of tumor growth was mainly due to targeting of the tumor vasculature. During the study period no difference in body weight between the CD99 vaccinated and control-vaccinated mice was observed in all three different vaccination studies performed (Supplementary Figure 2C). This suggests that vaccination against CD99 is well-tolerated and safe. In addition, we investigated if the antibodies induced against CD99 would recognize native CD99 in tissue. Therefore, we stained tissue of Os-P0107 osteosarcoma tumors derived from TRXtr-vaccinated mice with serum of TRXtr-mCD99 (CD99) vaccinated mice or of control (TRXtr) vaccinated mice. A specific staining of CD99 in tumor tissue was observed as indicated by the arrows in the upper right panel of Supplementary Figure 4A and the arrowheads in the lower right panel (Supplementary Figure 4A).

### Vaccination Affects the Tumor Vasculature

Osteosarcoma tumor tissue of the first vaccination study (study I) was stained for the vascular marker CD31 to determine the effect of vaccination against CD99 on the tumor vasculature (Figure 2E, upper panels). Vaccination with TRXtr-mCD99 seemed to have an effect on the morphology of the vasculature of osteosarcoma tumors (Figure 2E, upper panels). More specifically, tumors retrieved from TRXtr-mCD99 vaccinated mice (CD99, red bars) were found to have a significantly lower vessel density (^*^P < 0.05) and lumen count (^*^P = 0.05) compared to tumors retrieved from control vaccinated mice (TRXtr, blue bars) (Figure 2G). In the CD99 low CT26 tumor model also a significantly lower vessel density (^*^P < 0.05) was found in tumors of CD99 vaccinated mice (Figures 2J,K).

Furthermore, we stained the osteosarcoma tumors for both the vessel marker CD31 and the pericyte marker Desmin to study the effect of vaccination on vessel functionality. As illustrated in Figure 2E (lower panels) and Figure 2H, a significantly lower pericyte coverage was found in TRXtr-mCD99 vaccinated tumor tissue (red bars) compared to TRXtr vaccinated tumor tissue (blue bars) (^*^P < 0.05, Figure 2H, left panel). Furthermore, tumors of CD99 vaccinated mice were found to have more vessel without pericytes than control vaccinated mice (^*^P < 0.05, Figure 2H, right panel).

### Induction of a Humoral Immune Response Against CD99 Is Safe

During the experimental period of 14 weeks of the tumor growth study we did not observe any toxicity of the TRXtr-mCD99 vaccine as addressed by body weight or macroscopic and behavioral characteristics between CD99 vaccinated and control-vaccinated mice. However, to further investigate the safety of the vaccine we vaccinated healthy C57BL/6 mice against CD99 and monitored their body weight over a period of 45 weeks (Figure 2L). The mice were kept with high anti-CD99 antibody levels during the whole study period. Once the anti-CD99 antibody level dropped below 50% of the starting level (the antibody level after the third vaccination) the mice were re-vaccinated. Control mice were vaccinated with the TRXtr protein. During the whole study period we did not observe any difference in body weight between the CD99 group and the control vaccinated mice (Figure 2L). In addition, all except one mouse in the CD99 group, which was lost to follow-up at week 35, were healthy during the whole study period. We also looked at the morphology of the organs of CD99 vaccinated and control mice, but did not find any changes in tissue morphology after vaccination against CD99 (Figures 2M,N; Supplementary Figures 2D, 3). All together these observations indicate that vaccination against CD99 is safe.

### A Distinct Human CD99 Isoform Is Present in Activated Endothelial Cells

**Figure 3:**
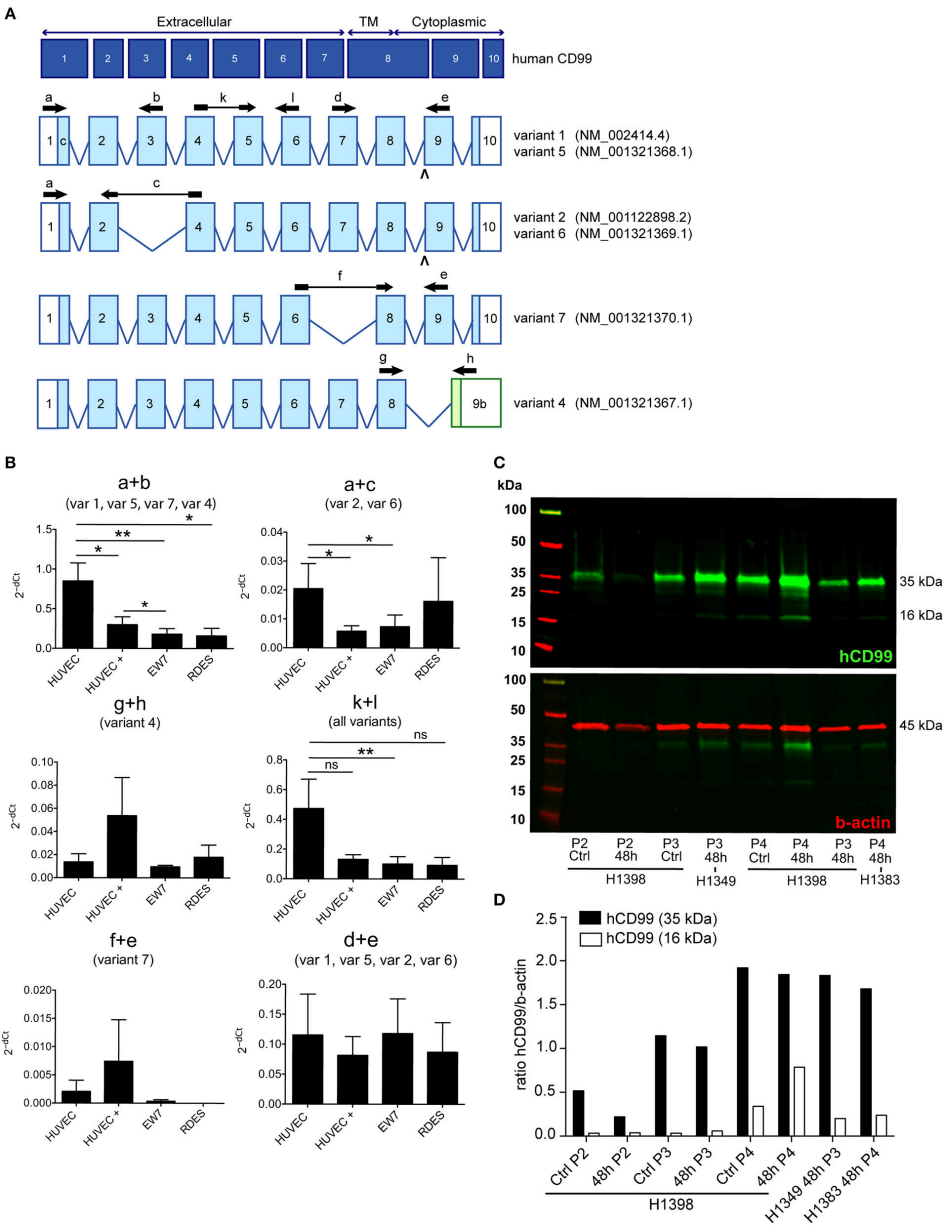
A distinct human CD99 isoform is present in activated endothelial cells. **(A)** Schematic representation of the human CD99 molecule and its exons (dark blue). The extracellular part (extracellular), the transmembrane region (TM) and the cytoplasmic domain (cytoplasmic) are indicated. Depicted in light blue are the different human CD99 isoforms (variants) retrieved from the NCBI database (CD99 molecule *Homo sapiens*, Gene ID: 4267). Accession numbers of the individual isoforms are indicated in brackets. Light blue exons are protein coding; UTRs are depicted in white. The inverted “v” below exon 9 indicates the presence of an additional alanine at the start of the exon and thereby a different variant (var) (variant 5 or 6). RT-qPCR-primers used for identification: the primer pair k + l detects all human CD99 variants (pan; total CD99). Primer pair d + e identifies variant 1, 5, 2, and 6. Primer pair a + b was used to detect the full-length CD99 isoform (var 1, var 5) and the variants 7 and 4. Primer pair a + c detects variant 2 and 6, which lack exon 3 in the extracellular domain. Primer pair f + e was used to identify variant 7, lacking exon 7. Primer pair g + h detects the truncated CD99 isoform (var 4, isoform **(C)**. **(B)** In activated HUVEC (HUVEC +; *n* = 9), EW7 (*n* = 11) and EWS-RDES (RDES; *n* = 3) relative expression (2^−dCt^) of total CD99 is downregulated on mRNA level (k + l; all variants). For native HUVEC (HUVEC; n = 6) vs. EW7 this is statistically significant (^**^P < 0.01) and for HUVEC vs. activated HUVEC (HUVEC +) [*P* = 0.0905; k + l)] and RDES (*P* = 0.0833; k + l) there is a trend toward significance. The observed difference of a + c between native and activated HUVEC (^*^P < 0.05) and EW7 (^*^P < 0.05) is due to that there is more total CD99 present (k + l primers) in native HUVEC compared to activated HUVEC. The ratio a + c/k + l is similar for both cell types (HUVEC = 0.04327756; HUVEC + = 0.0440165). A higher signal for the a + b primer pair is observed in native HUVEC compared to activated HUVEC+ (^*^P < 0.05), EW7 (^**^P < 0.01) or RDES (^*^P < 0.05). However, the ratio a + b/k + l is similar for all cell types (Table 7). The main isoform present in growth factor activated HUVEC (HUVEC+) is variant 4 (the short CD99 isoform, isoform **(C)**, identified by primer pair g + h. **(C)** Western blot of cell lysates from control treated HUVEC (Ctrl) and HUVEC grown for 48 h in culture medium supplemented with 1% serum (48 h). Different passages (P2–P4) of the same HUVEC (H1398) were used and P3 and P4 of different HUVEC (H1349 and H1383, respectively). The upper panel shows CD99 expression in the cell lysates (green). A protein band of 35 kDa can be observed in all lysates, with increasing passages a 16 kDa band is induced. In the lower panel the same Western blot is shown as in the upper panel, but now stained for β-actin (45 kDa; red), which was used as a loading control. **(D)** Data of the Western blot were quantified with ImageJ software. Expression of CD99 is induced in activated HUVEC; cells in higher passages express more CD99 (35 kDa band, black bars). In addition, there is a trend toward induction of expression of the 16 kDa band (white bars, the short CD99 isoform; isoform **(C)** with higher passage and by starvation of the cells.

In literature two different human CD99 isoforms have been described (27). A long full-length isoform (185 amino acids, 32 kDa, variant I; Supplementary Figure 1C) and a short truncated isoform (161 amino acids, 28 kDa, variant II), lacking most of the cytoplasmic domain. In the NCBI database six different protein coding human CD99 splice variants are suggested (Gene ID: 4267) (30) (Figure 3A). To distinguish the different human CD99 isoforms, as described in the NCBI database (Table 6), we designed RT-qPCR primers specific for the different splice variants (Figure 3A and Table 2). In Supplementary Figure 5 the different human CD99 splice variants retrieved from the NCBI database (Supplementary Figure 5A) and the Ensembl database (Supplementary Figure 5B) and their protein sequences (Supplementary Figure 5C) are depicted.

**Table 6:** Human CD99 isoforms listed in the NCBI database.

**Gene**	**Variant**	**Protein**	**Isoform**	**Size (kDa)**
NM_002414.4	Variant 1	NP_002405.1	Isoform A	18.8
NM_001122898.2	Variant 2	NP_001116370.1	Isoform B	17.1
NM_001321367.1	Variant 4	NP_001308296.1	Isoform C	16.0
NM_001321368.1	Variant 5	NP_001308297.1	Isoform D	18.8
NM_001321369.1	Variant 6	NP_001308298.1	Isoform E	17.0
NM_001321370.1	Variant 7	NP_001308299.1	Isoform F	17.2

In activated HUVEC (HUVEC+; *n* = 9), EW7 (*n* = 11), and EWS-RDES (RDES; *n* = 3) expression of total CD99 (k + l primers; all variants) is downregulated on mRNA level (Figure 3B). For native HUVEC (HUVEC; *n* = 6) vs. EW7 this is statistically significant (^**^P < 0.01) and for HUVEC vs. activated HUVEC (ns *P* = 0.09) and RDES (ns *P* = 0.08) there is a trend toward significance. The difference in expression found with the a + b and a + c primers in native HUVEC is due to high expression of total CD99 (k + l primers) in native HUVEC compared to the other cell types; as determined by the ratio of a + b/k + l and a + c/k + l (Table 7). On mRNA level however, there is a trend toward a higher expression of the short human CD99 isoform variant 4 (g + h primers; Table 7).

**Table 7:** Ratio variant specific primers vs. total CD99 (k + l primers).

	**Ratio a + b/k + l**	**Ratio a + c/k + l**	**Ratio g + h/k + l**
HUVEC	1.640392	0.043278	0.0290038
HUVEC+	2.280937	0.044017	0.408618
EW7	1.794606	0.072968	0.0949811
RDES	1.736055	0.175196	0.1944184

Western blot was performed on cell lysates of growth factor activated and serum starved HUVEC to determine if the short CD99 isoform could be detected on protein level. In activated HUVEC, after several passages (>P2) in culture, an additional protein band around 16 kDa can be observed next to the 35 kDa band of human CD99 (Figure 3C, green bands, upper panel). β-actin was used as a loading control (Figure 3C, 45 kDa, red band, lower panel). We quantified the ratio of the human CD99 bands (35 kDa, black bars; and 16 kDa, white bars) and β-actin with ImageJ software (Figure 3D, Table 8) and found that expression of CD99 is induced in activated HUVEC; cells in higher passages express more CD99 (35 kDa band, black bars). In addition, there is a trend toward induction of expression of the 16 kDa band (white bars, the short CD99 isoform; isoform C) with higher passage and after starvation of the cells for 48h.

**Table 8:** Western blot quantification hCD99 expression in HUVEC.

	**Band area** **b-actin**	**Band area** **hCD99 (35 kDa)**	**Band area** **hCD99 (16 kDa)**	**Ratio hCD99** **(35 kDa)/b-actin**	**Ratio hCD99** **(16 kDa)/b-actin**
H1398 Ctrl P2	6835.426	3532.154	239.092	0.51674234	0.03497836
H1398 48 h P2	3108.770	686.284	116.950	0.22075741	0.03761938
H1398 Ctrl P3	5808.891	6647.004	199.971	1.14428107	0.03442499
H1398 48 h P3	5428.841	9946.832	1095.820	1.83222017	0.20185156
H1398 Ctrl P4	4948.770	9503.489	1683.820	1.92037395	0.34025020
H1398 48 h P4	5567.255	10257.631	4373.962	1.84249347	0.78565864
H1349 48 h P3	4026.698	4097.447	239.092	1.01756998	0.05937669
H1383 48 h P4	4014.113	6741.518	956.577	1.67945397	0.23830346

Peripheral blood mononuclear cells (PBMC) of healthy volunteers only express CD99 at low levels (Supplementary Figure 4B). The main CD99 variants detected in PBMC were variant 1, variant 5, variant 7, and variant 4 (primer pair a + b), of which variant 7 (primer pair f + e) was basically undetectable (data not shown).

We also isolated mRNA from human colorectal carcinoma and renal cell carcinoma and matching healthy tissue, but were not able to determine any conclusive CD99 isoform expression pattern. This is most likely due to the fact that only 1–2% of all cells present in a tumor are endothelial cells and therefore it is very difficult to pick up specific splice variants.

These results indicate that CD99 splicing is tissue specific and provide an opportunity for specific targeting of CD99 isoforms in human tumor vasculature.

## Discussion

It was previously described that CD99 is overexpressed in inflamed vasculature. Our study demonstrated that CD99 is also overexpressed in the vasculature of solid tumors. CD99 is also known as a marker of Ewing's sarcoma (EWS). A therapeutic vaccination approach against CD99 could be of benefit for EWS patients. In addition, vaccination against CD99 could be used to treat other solid tumors by means of targeting the tumor vasculature.

For construction of the vaccine fusion protein TRXtr-mCD99, we used the protein sequence of the extracellular part of murine CD99 as described in Bixel et al. (36). We show that it is possible to induce a polyclonal antibody response against the self-antigen CD99 in immunocompetent mice by vaccination. Vaccination induced high levels of anti-mCD99 antibodies in the sera of the mice. This confirms the findings of previous studies using the same vaccination strategy for the induction of antibodies against different self-antigens (23, 24, 37).

In three independent studies a significantly smaller tumor volume was measured in the TRXtr-mCD99 vaccinated mice compared to control vaccinated mice. In this context, it is important to keep in mind that our vaccination strategy induces a polyclonal antibody response that is much more effective in inducing immune system activation than monocloncal antibodies (38). A polyclonal antibody response induces antibody-dependent cellular cytotoxicity (ADCC) where the antibodies function as a recognition and binding site for non-specific toxic cells like natural killer cells and macrophages. It also induces complement-dependent cytotoxicity (CDC) where the antibodies activate the complement system which leads to the formation of the membrane attack complex (MAC) and subsequent lysis of the target cell (39, 40).

In the CT26 model only low levels of CD99 are expressed by the tumor cells as compared to the osteosarcoma model. However, in the CT26 model also a significantly lower vessel density was observed in tumors of CD99 vaccinated mice. It is therefore likely that tumor growth inhibition in the CT26 model is mainly due to targeting of the tumor vasculature by the CD99 vaccine. The osteosarcoma model highly expresses CD99 and therefore inhibition of tumor growth in this model is due to targeting of both the tumor vasculature and the tumor cells, which leads to a more pronounced anti-tumor effect.

Anti-CD99 antibodies induced by the TRXtr-mCD99 were able to detect native CD99 in osteosarcoma tumor tissue. However, a lot of background staining was observed when the sections were stained with serum from CD99 vaccinated mice. This can be explained by the fact that staining mouse tissue with murine antibodies is difficult. We therefore used a F(ab) fragment to prevent non-specific binding of the serum. However, with this approach still a lot of background staining was observed. We have considered to purify IgG from mouse serum or to specifically purify the anti-CD99 antibodies in the serum with antigen, but we did not have sufficient mouse serum to do so.

After vaccination against CD99 we found more vessels without pericytes in the osteosarcoma tumors. This indicates that vascular targeting leading to vessel destruction occurs rather than vascular normalization after which a higher pericyte coverage is expected (41, 42). Vascular normalization is characterized by neutralization of growth factors, such as vascular endothelial growth factor (VEGF) (43). Neutralization of VEGF results in a more quiescent vasculature with more pericyte coverage and improved vascular flow. Vascular targeting on the other hand, leads to killing of the tumor endothelial cells, since these are attacked and removed by the immune system. This would explain our observations of a lower number of vessels with a lumen and pericytes in the tumors of CD99 vaccinated mice. As, the target CD99 is a transmembrane molecule tissue bound frustrated phagocytosis will occur (23) and not only the endothelial cells will be destroyed but everything in their vicinity as well, including pericytes.

No toxicity of the vaccination against CD99 was observed. In the tumor growth studies, no weight loss of the mice occurred during the study period. In addition, we monitored the body weight and health condition of CD99 vaccinated mice, with constantly high anti-mCD99 antibody levels in their sera, and control vaccinated mice, for a period of 45 weeks. In this study one mouse (CD99#2) was lost to follow-up. Considering the good health of all other CD99 vaccinated mice, this was probably due to non-treatment related conditions. We scrutinized the morphology of the organs of control and CD99 vaccinated mice and did not observe any changes in tissue morphology related to the presence of anti-mCD99 antibodies, neither based on vessel staining (CD31) of kidney vasculature and hematoxylin eosin staining of heart, lung, kidney, and liver. For the mouse that was lost to follow-up both the tissue and vessel morphology were normal/comparable to control vaccinated mice, implying that the loss of the mouse was probably not due side-effects of the vaccine. Taken together, our data suggest that vaccination against CD99 is safe, and provides a vascular targeting approach that lacks the risk of current angiostatic approaches for running into drug-induced resistance (44, 45).

Expression of CD99 was upregulated in growth factor activated HUVEC, resembling tumor endothelial cells, as determined by Western blot analysis. In addition, CD99 expression on cultured endothelial cells was induced by nutrient deprivation, which suggests that expression of CD99 in the tumor vasculature is most likely regulated by microenvironmental stress. We did not investigate if distinct CD99 isoforms are induced by starvation. To define which human CD99 isoform is the main variant present in the tumor vasculature we would need to perform a RT-qPCR on mRNA isolated from by flow cytometry sorted tumor endothelial cells. In literature, two distinct human CD99 isoforms have been described (27, 46). The full-length isoform (variant I, 32 kDa) and the truncated isoform (variant II, 28 kDa). Variant II includes a premature stop-codon caused by an insertion in the cytoplasmic domain and therefore lacks most of the cytoplasmic domain and is thought to be non-functional. The murine CD99 only shows 46% homology with human CD99 and resembles the human short isoform (28). However, if CD99 has the same function in mouse as in humans is unclear (29).

Currently, six different protein coding human CD99 isoforms are described in the NCBI database. We found contradictory results of CD99 expression on mRNA level and protein level in growth factor activated HUVEC. On mRNA level expression of CD99 seems to be downregulated in activated HUVEC, whereas on protein level CD99 expression is upregulated in activated HUVEC. On mRNA level the main CD99 isoform identified is variant 4, the short CD99 isoform, lacking part of the cytoplasmic domain. This is consistent with appearance of a 16 kDa protein band in higher passages of HUVEC and after starvation of the cells. If the 16 kDa protein band is a true splice variant of human CD99 is difficult to determine, since the anti-human CD99 antibody that we used is a polyclonal antibody that cannot distinguish between different splice variants. The human CD99 protein is highly O-glycosylated (47) and starvation of cells changes their glycosylation pattern (48), therefore the 16 kDa band observed in the Western blot might be nonglycosylated CD99. Also, in the Western blot a protein band between 35 and 25 kDa can be observed in some of the HUVEC cell lysates. We did however not further investigate if this could be a human CD99 splice variant.

In conclusion targeting of CD99 by vaccination inhibits tumor growth in different murine tumor models and is safe. Human CD99 is overexpressed in HUVEC and expression of CD99 is induced in culture and by nutrient deprivation. Also, a distinct human CD99 isoform is induced under these conditions, which is distinct form the isoforms expressed by EWS and healthy PBMC. These observations provide an opportunity for specific targeting of CD99 isoforms in human tumor vasculature.

## Data Availability

All datasets generated for this study are included in the manuscript and/or the Supplementary Files.

## Author Contributions

EH designed research, performed experiments, analyzed data, and wrote the manuscript. IvdW, LF, LS, PvdL, and HH performed experiments, analyzed data, and edited the manuscript. JvB designed research, analyzed data, and edited the manuscript. VT designed research and edited the manuscript. AC performed experiments and edited the manuscript. AG designed research and wrote the manuscript.

### Conflict of Interest Statement

The authors declare that the research was conducted in the absence of any commercial or financial relationships that could be construed as a potential conflict of interest.

## Supplementary Material

Supplementary Figure 1 Expression of CD99 in the tumor vasculature and vaccination strategy. **(A)** Different tumor types and normal healthy tissues stained for CD99 obtained from the Human Protein Atlas (scale bar 10 μm) and own staining of Ewing's sarcoma (EWS, right panels; upper panel scale bar 10 μm; lower panel scale bar 50 μm). All tumor types show staining of CD99 in the tumor vasculature (in hepatic cancer and EWS indicated by arrow heads). **(B)** Illustration of the vaccination strategy required for breaking self-tolerance. (1) The fusion protein (TRXtr-mCD99_extracellular_) mixed with a potent adjuvant is injected s.c. or i.m. (2) Antigen presenting cells (pink) will take up the fusion protein, digest it into self (mCD99) and foreign (TRXtr) peptides and present these peptides on their MHC class 2. (3) Foreign peptides are recognized by T-helper cells (Th, green) and these become activated. The presented self-peptides will not be recognized by the Th cells, since it is believed that all self-reactive T-cells are deleted in the thymus during development. (4) Auto-reactive B-cells (blue), existing in the circulation, recognize the self-part of the fusion protein via their B-cell receptor, internalize the fusion protein, and present self- and foreign peptides via MHC class II. The by the foreign peptides activated T-helper cells will now activate the auto-reactive B-cells, since they present the same foreign peptides. (4) The activated B-cells undergo clonal expansion and produce anti-self (mCD99) antibodies. By this means a polyclonal antibody response against the mCD99 is induced. **(C)** Schematic representation of the human CD99 protein: signal peptide (amino acids (aa) 1–22 (signal; white); extracellular domain aa 23–122 (extracellular; green); transmembrane region (TMR; pink) aa 123–147; cytoplasmic domain aa 148–185 (Cyto; orange). Retrieved from the uniport data base (UniProtKB—P14209 (CD99_human). **(D)** Illustration of the pET21a expression vector encoding TRXtr-mCD99. The TRXtr-mCD99_extracellular_ DNA sequence was inserted between the restriction sites *NdeI* and *XhoI* into the multiple cloning site (MSC). Protein expression is under the control of the IPTG-inducible T7*lac* promoter. Amp, *Ampicillin resistance gene*.

Supplementary Figure 2 Additional data osteosarcoma study I and II, CT26 study and long-term follow-up study. **(A)** Antibody titers of anti-CD99 antibodies in the sera of TRXtr (*n* = 5; left panel) and TRXtr-mCD99 (*n* = 10; CD99; middle and right panel) vaccinated mice at time point 9 weeks of study I Os-P0107 (C3H mice). TRXtr vaccinated mice are devoid of anti-CD99 antibodies. **(B)** Anti-mCD99 antibody levels in the sera of the C3H mice (Os-P0107 model) at different time points (weeks) of study II (*n* = 5 mice per group). **(C)** Body weight of CD99 vaccinated (CD99; red) and control vaccinated mice (TRXtr; blue) of the osteosarcoma study I and II (left and middle panel) and the CT26 study (right panel). No difference in body weight between the treatment groups was observed in all three different studies. Values are depicted as mean ± SEM. [study I: TRXtr (*n* = 5); CD99 (*n* = 10); study II: TRXtr and CD99 (*n* = 5); CT26: TRXtr and CD99 (*n* = 4)] **(D)** Kidneys stained for CD31 (brown-reddish staining) of TRXtr-mCD99 (*n* = 5; CD99) and control vaccinated (*n* = 5; TRXtr) mice from the long-term follow-up study (time point 45 weeks). Tissues were counter stained with Mayer's hematoxylin (blue) (scale bar 50 μm). No difference in tissue morphology was found between TRXtr-mCD99 vaccinated and control vaccinated mice.

Supplementary Figure 3 Morphology of normal organs of TRXtr-mCD99 and TRXtr vaccinated mice of the long-term follow-up study. **(A)** Hematoxylin eosin staining of organs (heart, lung, kidney, liver) of TRXtr-mCD99 (*n* = 5; CD99) and control vaccinated (*n* = 5; TRXtr) mice from the long-term follow-up study (time point 45 weeks) (scale bar 35 μm). No difference in tissue morphology was found between TRXtr-mCD99 vaccinated and control vaccinated mice.

Supplementary Figure 4 Anti-mCD99 antibodies induced by the TRXtr-mCD99 vaccine recognize native CD99 in tumor tissue. **(A)** Os-P0107 tumor tissues from control vaccinated mice were stained with either serum derived from TRXtr-vaccinated mice (TRXtr, left panels) or TRXtr-CD99 vaccinated mice (CD99, right panels). The upper right panel shows specific staining of CD99 as indicated by the arrows. In the lower right panel specific staining for CD99 is indicated by the arrow heads. All sections show high background, because mouse serum was used on mouse tissue (upper panels, scale bars 35 μm; lower panels, scale bars 50 μm). **(B)** Relative expression (2^−dCt^) of human CD99 variants in peripheral blood mononuclear cells (PBMC) (*n* = 3; three different healthy donors). Only low levels of CD99 are present on mRNA level in PBMC (k + l primer pair). The main variants detected in PBMC are variant 1, variant 5, variant 7, and variant 4 (var 1, var 5, var 7, var 4) identified by primer pair a + b.

Supplementary Figure 5 Human CD99 splice variants. **(A)** Human CD99 variants described in the NCBI database Gene ID: 4267. **(B)** Human CD99 variants described in the Ensembl database Gene: CD99 ENSG00000002586. **(C)** Alignment of the protein sequences of the different human CD99 splice variants.
