# Supplementary figures and images for: Targeting Tumor Vascular CD99 Inhibits Tumor Growth
Source: Front Immunol. 2019 Apr 2;10:651. doi: 10.3389/fimmu.2019.00651 (PMC6455290; doi:10.3389/fimmu.2019.00651)

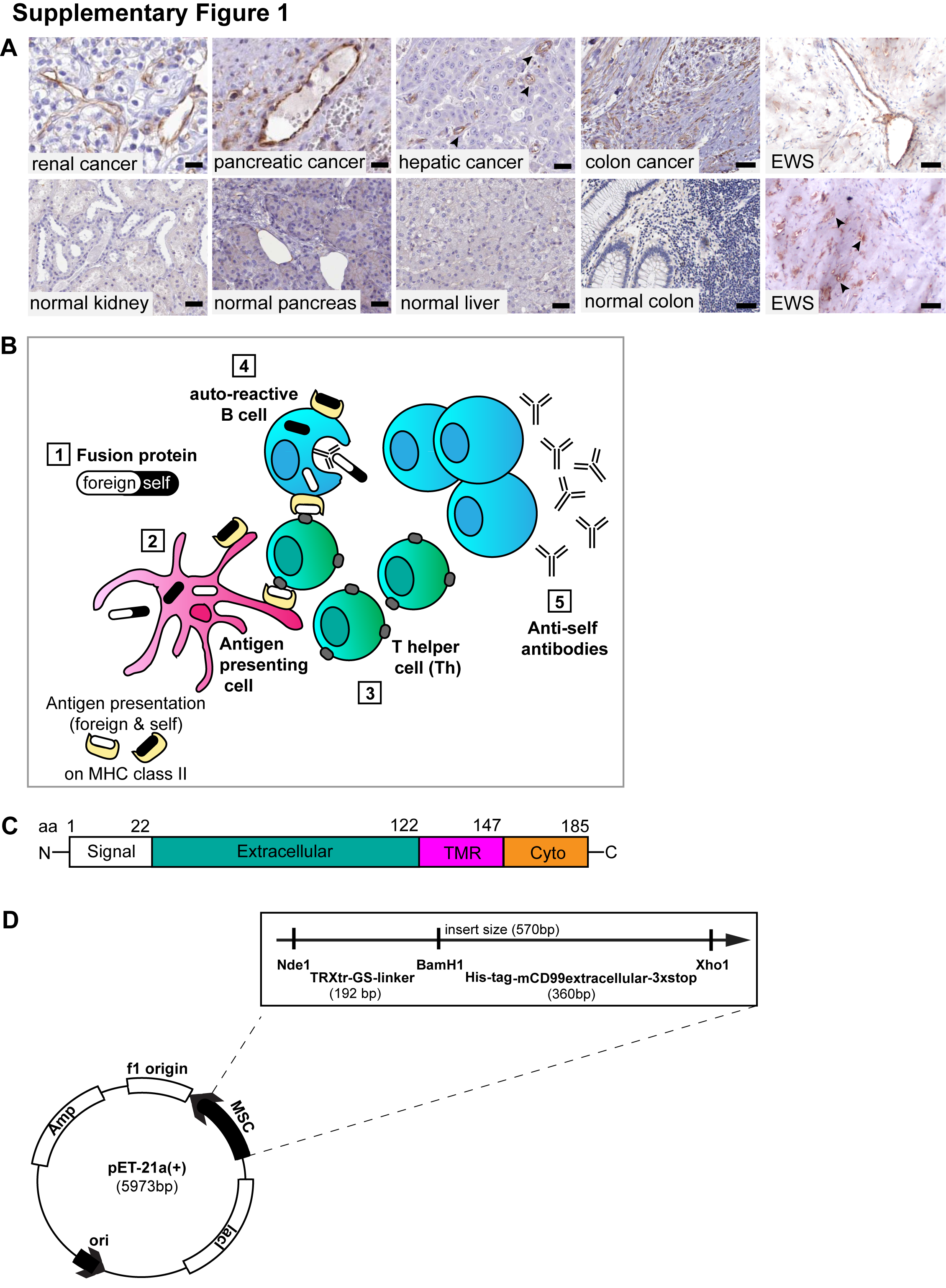

Supplement: Supplementary Figure 1 — Expression of CD99 in the tumor vasculature and vaccination strategy. (A) Different tumor types and normal healthy tissues stained for CD99 obtained from the Human Protein Atlas (scale bar 10 μm) and own staining of Ewing's sarcoma (EWS, right panels; upper panel scale bar 10 μm; lower panel scale bar 50 μm). All tumor types show staining of CD99 in the tumor vasculature (in hepatic cancer and EWS indicated by arrow heads). (B) Illustration of the vaccination strategy required for breaking self-tolerance. (1) The fusion protein (TRXtr-mCD99extracellular) mixed with a potent adjuvant is injected s.c. or i.m. (2) Antigen presenting cells (pink) will take up the fusion protein, digest it into self (mCD99) and foreign (TRXtr) peptides and present these peptides on their MHC class 2. (3) Foreign peptides are recognized by T-helper cells (Th, green) and these become activated. The presented self-peptides will not be recognized by the Th cells, since it is believed that all self-reactive T-cells are deleted in the thymus during development. (4) Auto-reactive B-cells (blue), existing in the circulation, recognize the self-part of the fusion protein via their B-cell receptor, internalize the fusion protein, and present self- and foreign peptides via MHC class II. The by the foreign peptides activated T-helper cells will now activate the auto-reactive B-cells, since they present the same foreign peptides. (4) The activated B-cells undergo clonal expansion and produce anti-self (mCD99) antibodies. By this means a polyclonal antibody response against the mCD99 is induced. (C) Schematic representation of the human CD99 protein: signal peptide (amino acids (aa) 1–22 (signal; white); extracellular domain aa 23–122 (extracellular; green); transmembrane region (TMR; pink) aa 123–147; cytoplasmic domain aa 148–185 (Cyto; orange). Retrieved from the uniport data base (UniProtKB—P14209 (CD99_human). (D) Illustration of the pET21a expression vector encoding TRXtr-mCD99. The TRXtr-mCD99e [file Image_1.TIF]

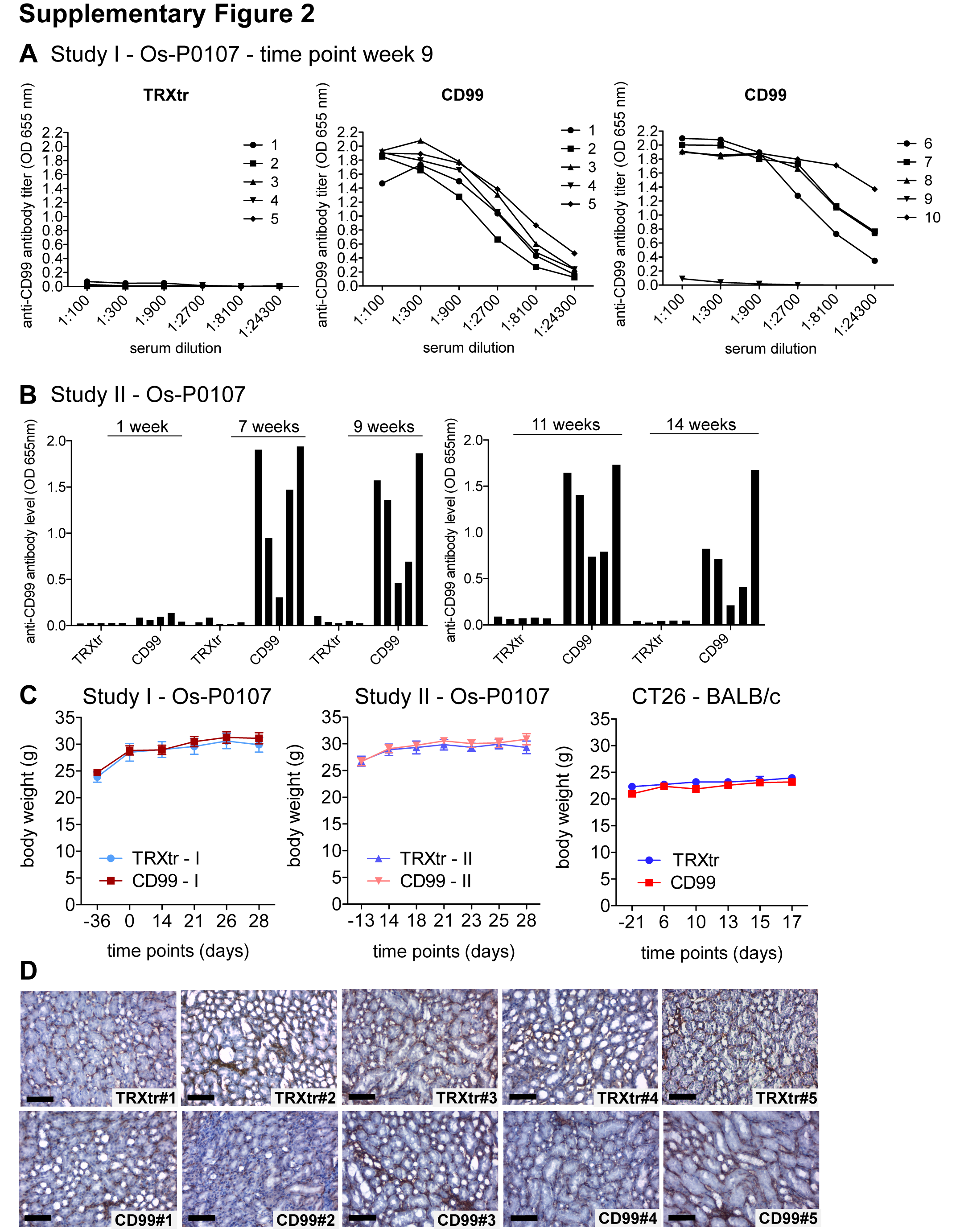

Supplement: Supplementary Figure 2 — Additional data osteosarcoma study I and II, CT26 study and long-term follow-up study. (A) Antibody titers of anti-CD99 antibodies in the sera of TRXtr (n = 5; left panel) and TRXtr-mCD99 (n = 10; CD99; middle and right panel) vaccinated mice at time point 9 weeks of study I Os-P0107 (C3H mice). TRXtr vaccinated mice are devoid of anti-CD99 antibodies. (B) Anti-mCD99 antibody levels in the sera of the C3H mice (Os-P0107 model) at different time points (weeks) of study II (n = 5 mice per group). (C) Body weight of CD99 vaccinated (CD99; red) and control vaccinated mice (TRXtr; blue) of the osteosarcoma study I and II (left and middle panel) and the CT26 study (right panel). No difference in body weight between the treatment groups was observed in all three different studies. Values are depicted as mean ± SEM. [study I: TRXtr (n = 5); CD99 (n = 10); study II: TRXtr and CD99 (n = 5); CT26: TRXtr and CD99 (n = 4)] (D) Kidneys stained for CD31 (brown-reddish staining) of TRXtr-mCD99 (n = 5; CD99) and control vaccinated (n = 5; TRXtr) mice from the long-term follow-up study (time point 45 weeks). Tissues were counter stained with Mayer's hematoxylin (blue) (scale bar 50 μm). No difference in tissue morphology was found between TRXtr-mCD99 vaccinated and control vaccinated mice. [file Image_2.TIF]

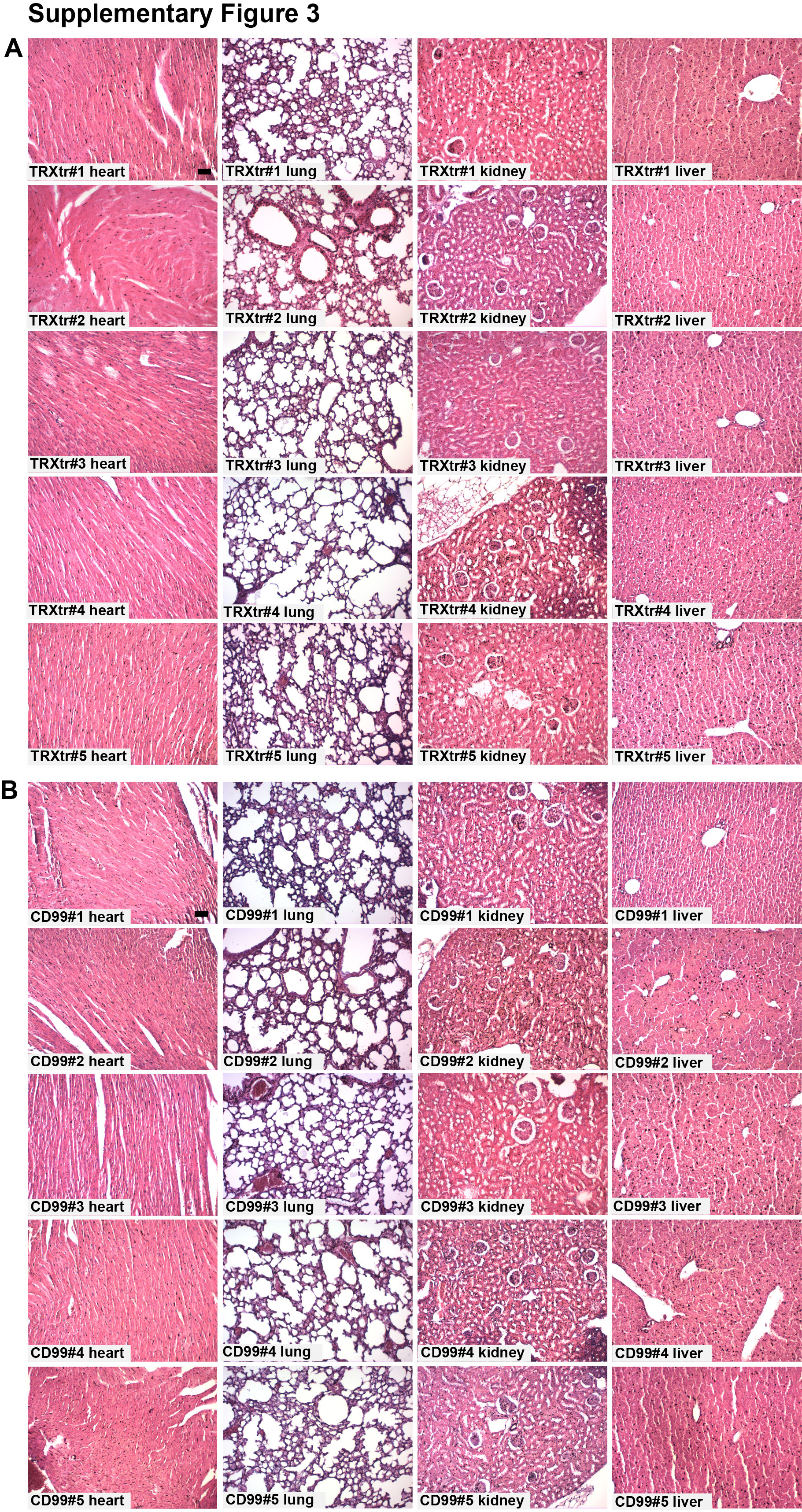

Supplement: Supplementary Figure 3 — Morphology of normal organs of TRXtr-mCD99 and TRXtr vaccinated mice of the long-term follow-up study. (A) Hematoxylin eosin staining of organs (heart, lung, kidney, liver) of TRXtr-mCD99 (n = 5; CD99) and control vaccinated (n = 5; TRXtr) mice from the long-term follow-up study (time point 45 weeks) (scale bar 35 μm). No difference in tissue morphology was found between TRXtr-mCD99 vaccinated and control vaccinated mice. [file Image_3.JPEG]

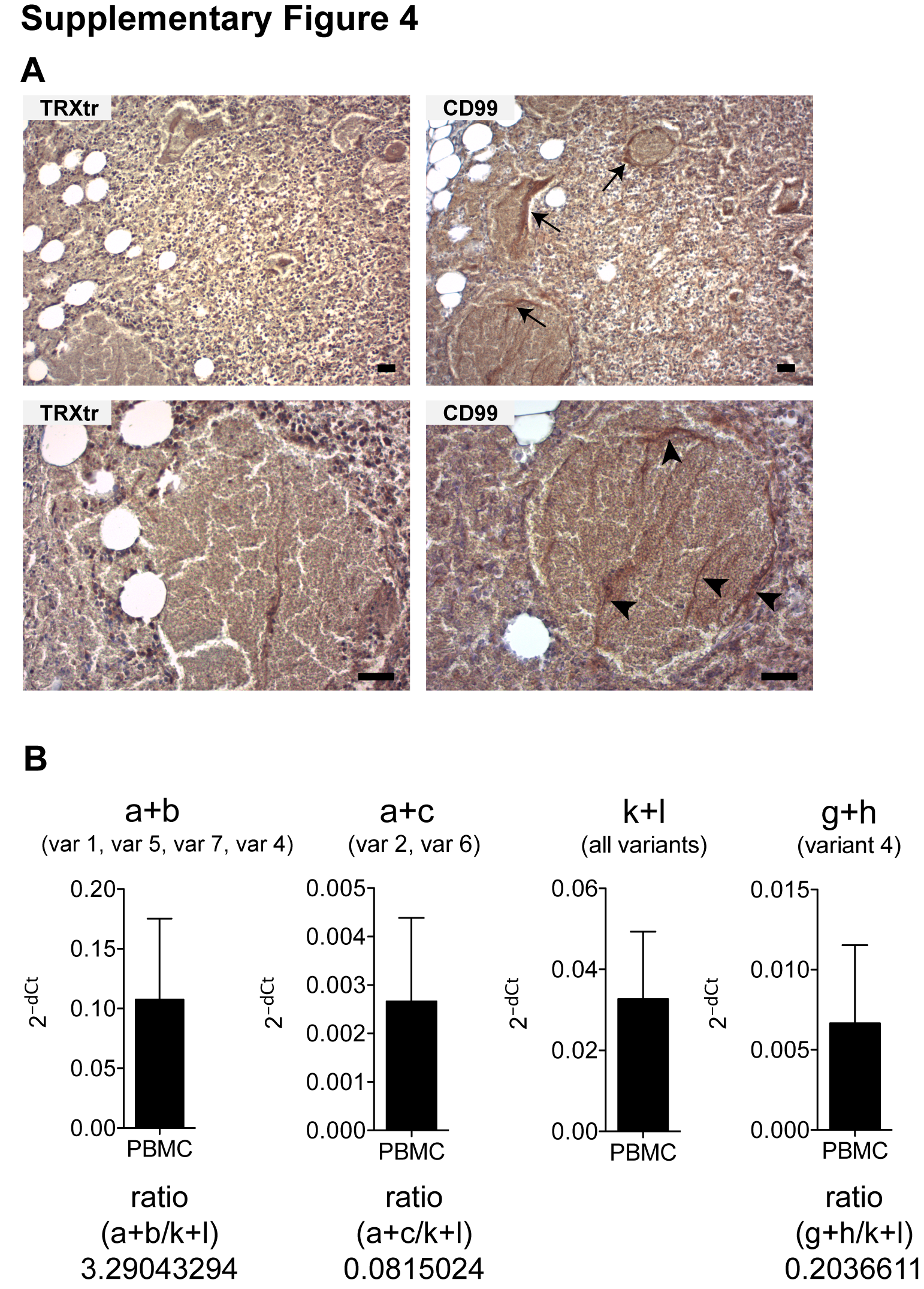

Supplement: Supplementary Figure 4 — Anti-mCD99 antibodies induced by the TRXtr-mCD99 vaccine recognize native CD99 in tumor tissue. (A) Os-P0107 tumor tissues from control vaccinated mice were stained with either serum derived from TRXtr-vaccinated mice (TRXtr, left panels) or TRXtr-CD99 vaccinated mice (CD99, right panels). The upper right panel shows specific staining of CD99 as indicated by the arrows. In the lower right panel specific staining for CD99 is indicated by the arrow heads. All sections show high background, because mouse serum was used on mouse tissue (upper panels, scale bars 35 μm; lower panels, scale bars 50 μm). (B) Relative expression (2−dCt) of human CD99 variants in peripheral blood mononuclear cells (PBMC) (n = 3; three different healthy donors). Only low levels of CD99 are present on mRNA level in PBMC (k + l primer pair). The main variants detected in PBMC are variant 1, variant 5, variant 7, and variant 4 (var 1, var 5, var 7, var 4) identified by primer pair a + b. [file Image_4.TIF]

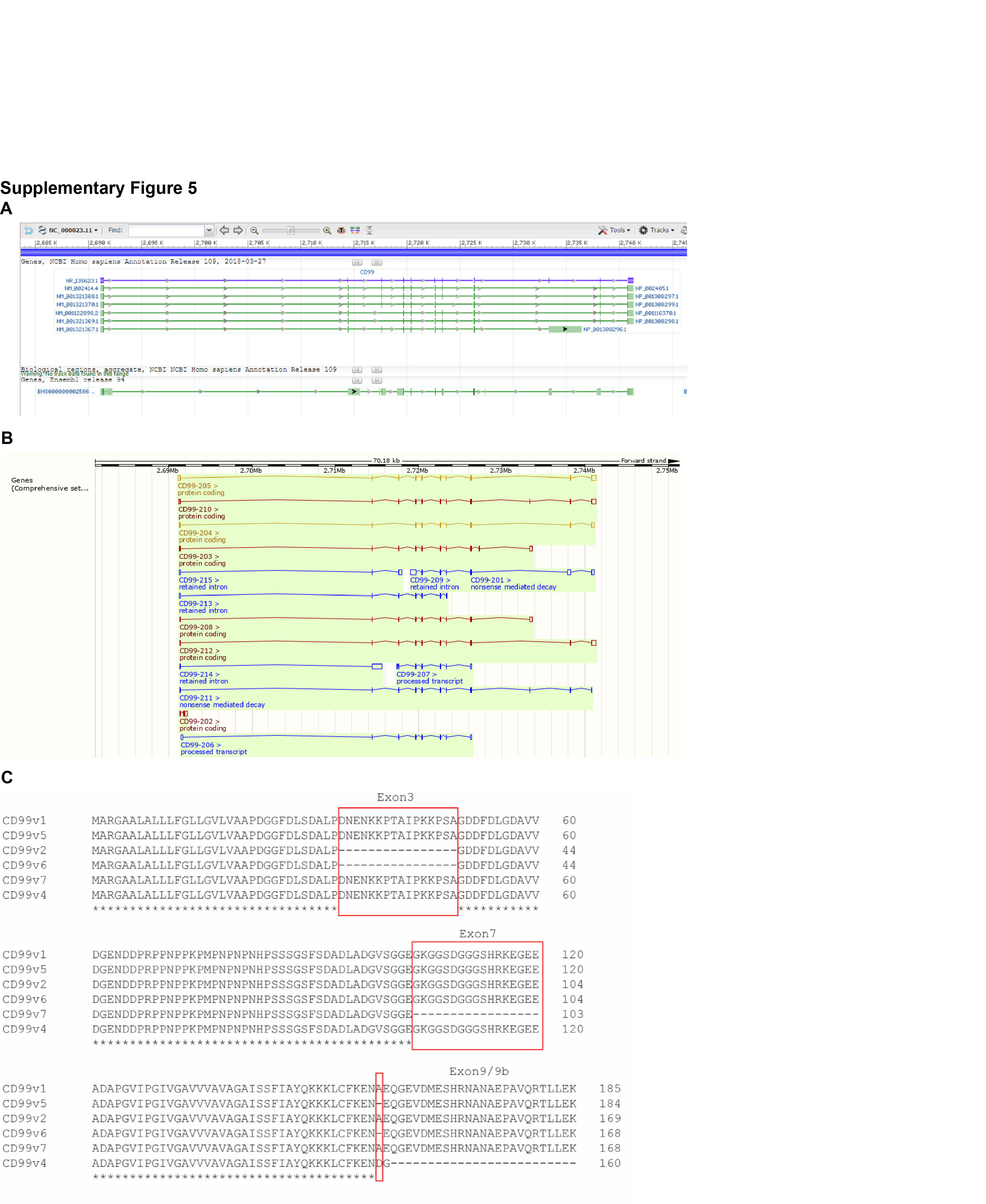

Supplement: Supplementary Figure 5 — Human CD99 splice variants. (A) Human CD99 variants described in the NCBI database Gene ID: 4267. (B) Human CD99 variants described in the Ensembl database Gene: CD99 ENSG00000002586. (C) Alignment of the protein sequences of the different human CD99 splice variants. [file Image_5.JPEG]
